# A resilient formin-derived cortical actin meshwork in the rear drives actomyosin-based motility in 2D confinement

**DOI:** 10.1038/ncomms9496

**Published:** 2015-09-29

**Authors:** Nagendran Ramalingam, Christof Franke, Evelin Jaschinski, Moritz Winterhoff, Yao Lu, Stefan Brühmann, Alexander Junemann, Helena Meier, Angelika A. Noegel, Igor Weber, Hongxia Zhao, Rudolf Merkel, Michael Schleicher, Jan Faix

**Affiliations:** 1 Anatomy III/Cell Biology, BioMedCenter, Ludwig-Maximilians-University, Grosshaderner Str. 9, Planegg-Martinsried, Germany; 2 Institute for Biophysical Chemistry, Hannover Medical School, Carl-Neuberg-Strasse 1, Hannover 30625, Germany; 3 Institute of Complex Systems, ICS-7: Biomechanics, Forschungszentrum Jülich GmbH, Jülich 52425 Germany; 4 Institute of Biotechnology, University of Helsinki, PO Box 56, Helsinki 00014, Finland; 5 Center for Biochemistry, Medical Faculty, University of Cologne, Köln 50931, Germany; 6 Division of Molecular Biology, Ruder Bošković Institute, Bijenička 54, Zagreb 10000, Croatia

## Abstract

Cell migration is driven by the establishment of disparity between the cortical properties of the softer front and the more rigid rear allowing front extension and actomyosin-based rear contraction. However, how the cortical actin meshwork in the rear is generated remains elusive. Here we identify the mDia1-like formin A (ForA) from *Dictyostelium discoideum* that generates a subset of filaments as the basis of a resilient cortical actin sheath in the rear. Mechanical resistance of this actin compartment is accomplished by actin crosslinkers and IQGAP-related proteins, and is mandatory to withstand the increased contractile forces in response to mechanical stress by impeding unproductive blebbing in the rear, allowing efficient cell migration in two-dimensional-confined environments. Consistently, ForA supresses the formation of lateral protrusions, rapidly relocalizes to new prospective ends in repolarizing cells and is required for cortical integrity. Finally, we show that ForA utilizes the phosphoinositide gradients in polarized cells for subcellular targeting.

## Abstract (web summary)

Amoeboid motility is driven by actomyosin-based contraction and exploits differences in the mechanical properties of the cortical cytoskeleton. Here the authors discover that mDia1-like formin A is responsible for generating a subset of actin filaments at the rear of *Dictyostelium* that suppresses lateral protrusions and blebbing during 2D-confined migration.

## Graphical abstract

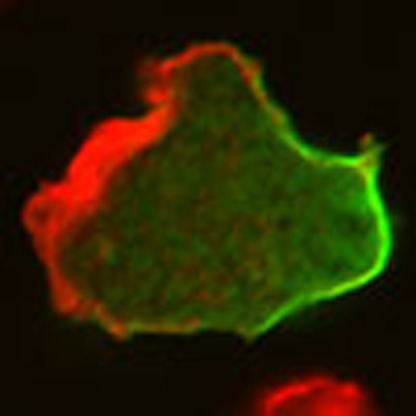

Cell migration is implicated in numerous processes in normal physiology and disease^1^,^2^. On the basis of their properties, eukaryotic cells can move by distinct modes of action. To delineate this heterogeneity, the behaviours of single cells have been subdivided into two main categories of mesenchymal or amoeboid migration modes depending on cell morphology, mechanisms of force generation, cytoskeleton organization and cell–substrate adhesion^1^,^3^. The rather slow mesenchymal mode of cell migration, as exemplified by fibroblasts, is characterized by strong cell–substrate adhesion, prominent stress fibres and extended formation of protruding lamellipodia or ruffles driven by Arp2/3 complex-mediated actin polymerization at the leading edge^4^. Fast amoeboid cell migration, as utilized by immune cells or *Dictyostelium* amoebae, is instead characterized by rounder shape, weaker adhesion, absence of stress fibres and formation of actin-rich pseudopods or hydrostatic pressure-driven blebs in their fronts and myosin-II-driven contractility in the rears^5^,^6^. Notably, these distinct motility modes are extremes of a broad spectrum characterized by smooth transitions. In addition, some cells, in particular cancer cells, exhibit plasticity and can switch from the mesenchymal to the amoeboid motility mode to drive invasion^1^.

The movement of cells is the final readout of multiple processes including actin assembly, adhesion and contractility, and entails the breaking of symmetry to generate a cell front and a cell rear along the axis in the direction of movement^7^. There is strong evidence that global actin–myosin network reorganization and non-muscle myosin-II-driven contraction initiate symmetry breaking by forming the imminent rear of the cell^8^, which restricts protrusions to the cell front. The transition from a semistable unpolarized state to a polarized migratory state can occur randomly, but can also be induced by mechanical stimulation, leading to an anisotropic distribution of the actomyosin system, which is subsequently sustained by positive-feedback loops^9^. Polarity can be additionally stabilized for instance in chemotactically migrating cells by extracellular cues^10^.

Cell membrane deformation is coupled to cortical tension and stiffness, membrane–cortex adhesion and hydrostatic pressure^3^,^11^. Micropipette aspiration (MPA) assays with polarized migrating *Dictyostelium* amoebae revealed easier deformability at the cell front as compared with the trailing edge, suggesting either weaker anchoring of the membrane to the underlying actin cytoskeleton or a less stiffer cortex in the leading edge^12^. Similar differences in the cortical properties have also been demonstrated for higher eukaryotes strongly suggesting that this disparity is a general requirement of actomyosin-driven or actomyosin-assisted cell migration^3^,^13^. The contractile actin cortex is a thin layer of bundled or crosslinked actin filaments, non-muscle myosin II and associated proteins beneath the plasma membrane of eukaryotic cells^11^,^14^. Assembly and contraction of this layer generates cortical tension and plays a central role in migration^7^, cell division^15^ and tissue morphogenesis^16^. Despite its significance, the assembly, structural organization, membrane attachment and mechanics of the actin-rich cortex are still not well understood.

Even though numerous proteins can promote actin assembly, Arp2/3 complex and formins are the major actin nucleators in cells^17^. Active Arp2/3 complex creates branches on the sides of existing mother filaments to generate a dense actin meshwork as exemplified by the actin architecture of the leading edge^18^. Formins instead nucleate and elongate unbranched actin filaments to form the cytokinetic ring, yeast cables or filopodial bundles^17^. A subgroup referred as diaphanous-related formins (DRFs) is tightly regulated. In their autoinhibited form, these proteins fold on themselves and are inactive. Binding of Rho-family GTPases to the N-terminal GTPase-binding domain (GBD) releases this autoinhibition and renders the proteins active. Both, Arp2/3 complex and different DRFs have been implicated in the formation of actin cortex in various cell types, although most of the obtained evidence was rather indirect^19^,^20^,^21^,^22^. More recent work convincingly demonstrated a major contribution of Arp2/3 complex and the DRF mDia1 in generation of the cortical actin cytoskeleton in M2 and HeLa cells^23^. As revealed by this study, Arp2/3 complex and mDia1 contributed equally to F-actin formation in the cortex but had different effects on cortical integrity, blebbing and cell division. While for instance mDia1 depletion arrested cell division, depletion or perturbation of Arp2/3 complex by itself did not. However, Arp2/3 complex perturbation potentiated the effect of mDia1 depletion, suggesting that mDia1 and Arp2/3 play different roles in cortex function.

In this study, we aimed to identify actin assembly factors that contribute to the formation and function of the rear cortex of polarized, migrating *Dictyostelium* cells. Our results show that ForA-generated actin filaments provide the basis of a resilient cortical shield that is strengthened by actin crosslinkers and IQ-motif-containing GTPase-activating protein (IQGAP)-related proteins. This actin compartment is recruited to the rear by phosphoinositide gradients and allows withstanding high actomyosin-based contractility and guides hydrostatic pressure to the front facilitating cell migration in a two-dimensional (2D)-confined environment. In addition, we reveal that this cortical network also suppresses the formation of unproductive lateral protrusions. Finally, our findings strongly suggest that ForA represents the functional homologue of mDia1 from higher eukaryotes.

## Results

### ForA localizes to the rear actin cortex of migrating cells

**Figure 1:**
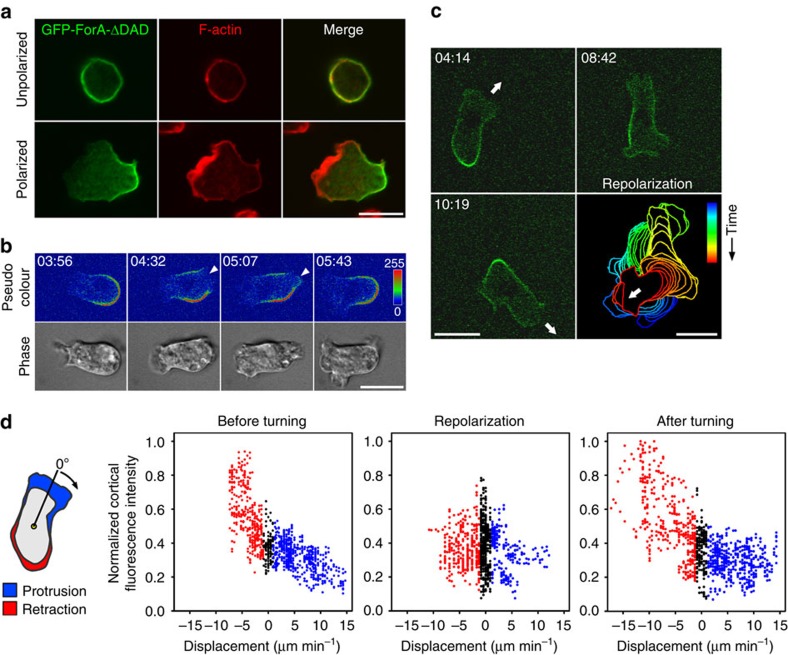
Cortical localization of ForA. (**a**) GFP-tagged ForAΔDAD expressed in *forA*^*−*^ cells localizes uniformly at the cortex of unpolarized cells but accumulates prominently at the trailing edge of polarized cells. Scale bar, 10 μm. (**b**) GFP-tagged ForAΔDAD, depicted a by pseudo-coloured heatmap, instantly withdraws from the cortex before formation of lateral protrusions (arrowheads). Time corresponds to min:s and refers to Supplementary Movie 3. Scale bar, 10 μm. (**c**) Relocation of active ForA in turning cells. The white arrow marks the direction of cell movement before and after repolarization and refers to Supplementary Movie 4. Colour-coded, stacked outlines of this cell during the turning event are shown in the low right corner. Scale bars, 10 μm. (**d**) ForA dynamically relocates to new prospective ends in repolarizing cells. Left: principle of radial displacement determination (for details see Methods). Plots show correlation between cortical ForA intensity and radial boundary displacement in retracting (red), non-dynamic (black) and protruding (blue) areas of three analysed cells before, during and after repolarization. In polarized, migrating cells ForA intensity was markedly increased in zones of high retraction at the rear and lower in protruding areas at the front. During repolarization, the edge displacements were smaller and unevenly distributed around the cell perimeter (see Supplementary Fig. 2c). Consistently, cortical ForA intensity was lower and homogenous at this stage.

Since the hydrostatic pressure-driven blebs are a hallmark of migrating *Dictyostelium* cells in confined environments^6^, and the loss of myosin II is detrimental for cell migration^24^, we hypothesized specific actin assembly factors to exist in the rear that contribute to actomyosin-driven contractility. As the Arp2/3 complex is predominantly localized in the cell front, we systematically screened for the localization of as yet uncharacterized *Dictyostelium* formins by expression of green fluorescent protein (GFP) fusion proteins. We identified active ForA, lacking its C-terminal diaphanous-autoinhibitory domain (ForAΔDAD), but not the autoinhibited full-length (FL) protein, to be strongly enriched in the rear cortex of migrating cells and the cleavage furrow of dividing cells, while it was evenly distributed in the cell cortex of unpolarized resting cells (Fig. 1a; Supplementary Fig. 1; Supplementary Movies 1 and 2). During random cell migration, vegetative *Dictyostelium* cells frequently generate multiple transient pseudopods along their periphery as opposed to developed chemotactically migrating cells that maintain a stable single front^25^. Interestingly, confocal time-lapse imaging of randomly migrating ForAΔDAD cells revealed that birth of a new extension in the rear was faithfully associated with concomitant disappearance of the formin (Fig. 1b; Supplementary Movie 3), demonstrating that migratory cell protrusions are exclusively formed from cortical regions devoid of ForA. ForAΔDAD also always dynamically altered its position in spontaneous front-to-tail transitions of sharply turning cells (exemplarily shown in Fig. 1c; Supplementary Movie 4). Global measurement of cortical ForA fluorescence intensity around the entire cell periphery in these cells before, during and after repolarization revealed ForA accumulation in retracting zones before and after changing the direction of movement, while ForA intensity was evenly distributed in repolarizing cells (Fig. 1d; Supplementary Fig. 2).

### ForA is regulated and promotes actin assembly

**Figure 2:**
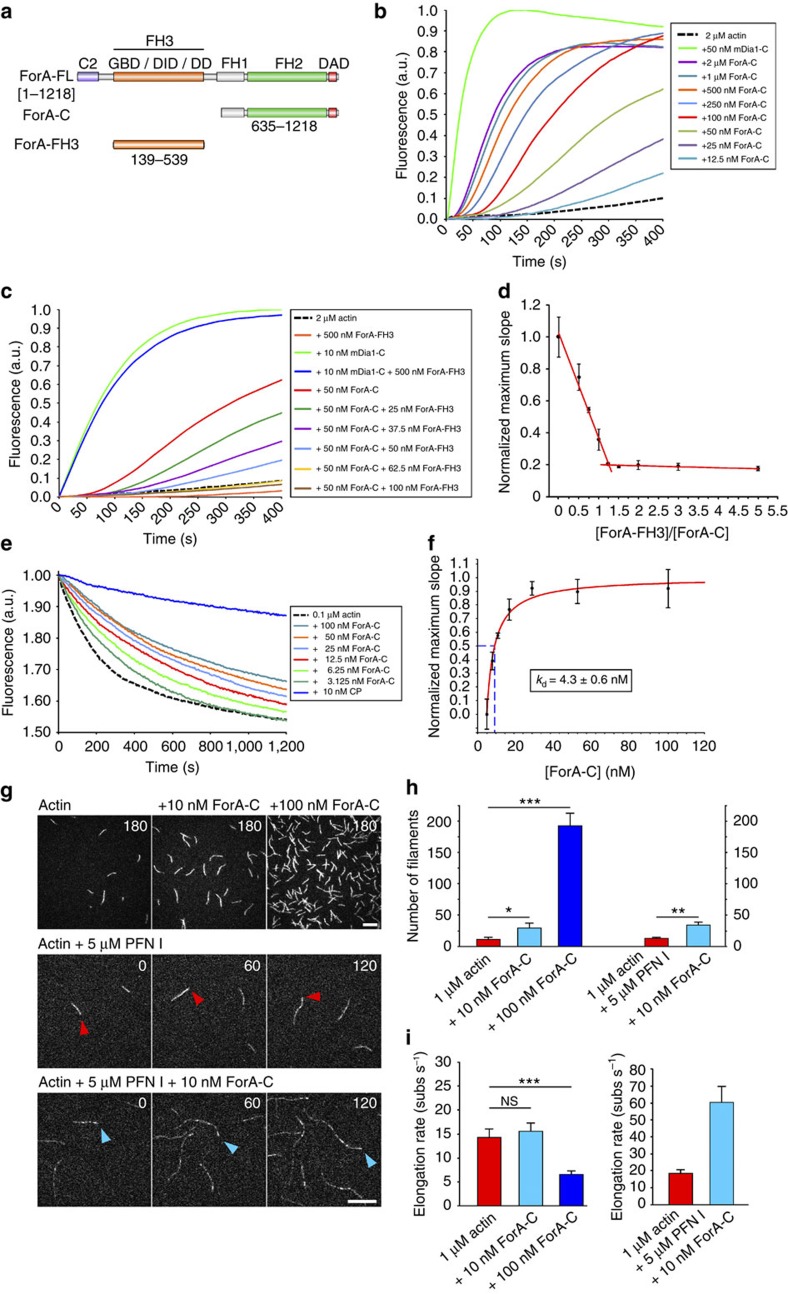
ForA is an autoinhibited formin that promotes actin assembly. (**a**) ForA constructs used for biochemical analyses. C2, C2 domain; GBD, GTPase-binding domain; DID, diaphanous inhibitory domain; DD, dimerization domain; FH, formin homology domain; DAD, diaphanous autoinhibitory domain. (**b**) ForA-C promotes actin assembly in pyrene assays in a concentration-dependent manner. A total of 50 nM mDia1-C served as a positive control. (**c**) ForA is autoinhibited. ForA-FH3 specifically inhibited actin polymerization of ForA-C in a concentration-dependent manner, but had no effect on mDia1-C. (**d**) Maximum slopes of the polymerization curves shown in **c** revealed high affinity of ForA-FH3 to ForA-C. Intersection of the solid lines (red) marks the molar ratio required for full inhibition indicating 1:1 stoichiometry. Bars represent mean±s.d. *n*=4. (**e**) ForA-C inhibits actin disassembly in dilution-induced depolymerization assays, demonstrating interaction with filament barbed ends. 10 nM capping protein (CP) served as a control for high-affinity binding to filament barbed ends. (**f**) Maximum slopes of the depolymerization curves as shown in **e** were fitted assuming one binding site at filament barbed ends and revealed an apparent *K*_d_ of 4.3±0.6 nM. Error bars represent mean±s.d., *n*=4. (**g**) Time-lapse micrographs of actin assembly visualized by *in vitro* TIRF microscopy. Upper panel displays number of nucleated filaments in the absence or presence of 10 or 100 nM ForA-C. Lower two panels illustrate barbed ends growth of a control filament (red arrowhead) and a ForA-C elongated filament (blue arrowhead) in presence of PFN I. Time is in seconds. Scale bars, 10 μm. (**h**) Quantification of nucleated filaments obtained from TIRF microscopy experiments as shown in **g**. Bars represent mean±s.d., *n*=3. ***P≤0.001, **P≤0.01, *P≤0.05 (*t*-test). (**i**) In the absence of PFN I, addition of 100 nM ForA-C (dark blue) led to significantly reduced elongation rates as compared with control (red). In the presence of PFN I, ForA-C-associated filaments (light blue) grew threefold faster than control filaments. Bars represent mean±s.d., *n*>20. ***P≤0.001, NS, not significant (Mann–Whitney *U*-test).

Canonical formins nucleate and elongate actin filaments, although their specific activities can be highly variable^26^. Thus, we analysed the biochemical properties of ForA *in vitro* after purification of a recombinant glutathione *S*-transferase (GST)-tagged C-terminal ForA fragment (ForA-C) encompassing five of the eight poly-proline stretches from its FH1 domain followed by all of its C-terminal residues (aa 635–1,218, Fig. 2a). After removal of the GST-tag, ForA-C was first analysed in pyrene actin assays in which it stimulated actin polymerization in a concentration-dependent manner (Fig. 2b). Addition of the N-terminal regulatory region (ForA-FH3; Fig. 2a) strongly inhibited actin assembly by ForA-C at a molar ratio of ∼1:1, indicating a strong interaction of the regulatory domains of this DRF (Fig. 2c,d). In dilution-induced depolymerization assays, ForA-C inhibited the depolymerization of actin filaments due to its association with filament barbed ends with a calculated apparent *K*_d_ of 4 nM (Fig. 2e,f). Next, we further examined its filament nucleation and elongation activities by single-filament total internal reflection fluorescence (TIRF) microscopy. Nanomolar concentrations of ForA-C noticeably stimulated the nucleation of filaments that subsequently grew with 60 subunits per second, in the presence of profilin I (PFN I), as compared with actin control filaments, which elongated with 18 subunits per second (Fig. 2g–i; Supplementary Movies 5 and 6), illustrating that ForA exerts prototypical formin activity. Collectively, these data therefore supported the notion of ForA contributing to the formation of cortical actin in the trailing edge.

### ForA is required for efficient motility in 2D confinement

**Figure 3:**
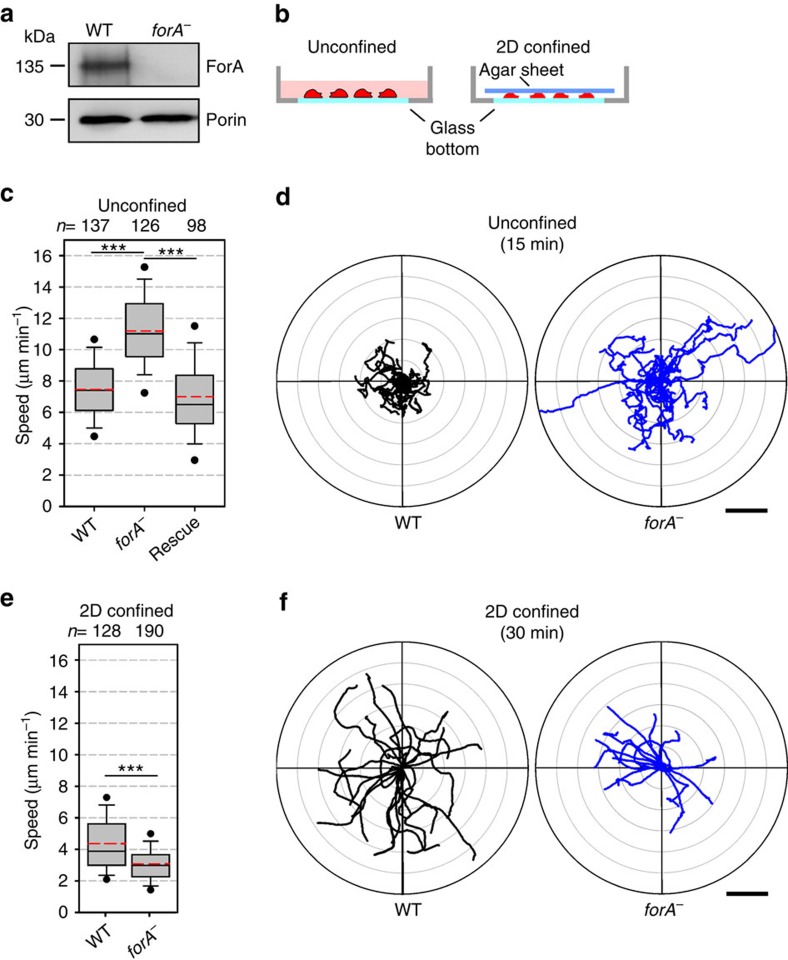
ForA effects motility in unconfined and 2D-confined environments. (**a**) Elimination of ForA was confirmed by immunoblotting. Porin was used as a loading control. (**b**) Scheme for analyses of cell migration in unconfined and 2D-confined settings. (**c**) Quantitative analysis of random cell migration in unconfined conditions revealed significantly faster motility of *forA*^*−*^ cells. (**d**) Cell trajectories of randomly migrating wild-type and *forA*^*−*^ cells of representative samples in radar plots illustrate faster motility of the mutant cells as compared with wild type (see Supplementary Movie 7). (**e**) Quantitative analysis of random cell migration in 2D-confined environments. When sandwiched between the glass surface and a thin sheet of agar, *forA*^*−*^ mutants showed diminished migration rates as compared with wild type (see Supplementary Movie 8). (**f**) Cell trajectories of randomly migrating wild-type and *forA*^*−*^ cells (20 each) of representative samples in radar plots illustrate compromised motility of *forA*^*−*^ cells in confinement (see Supplementary Movie 8). Boxes in **c** and **e** indicate 25–75 percentiles, the whiskers mark the 10^th^ and 90^th^ and the outliers the 5^th^ and 95^th^ percentile. Red dashed lines indicate mean values. *n*, number of tracked cells. ***P≤0.001 (Mann–Whitney *U*-test). Scale bars, 40 μm (**d**,**f**).

To assess the function of ForA in motility, we disrupted the *forA* gene by homologous recombination in AX2 wild-type cells. Successful elimination was confirmed by PCR and immunoblots using ForA-specific polyclonal antibodies (Fig. 3a). Intriguingly, the elimination of ForA markedly increased the speed of randomly migrating cells in unconfined environments to 11.2±2.5 μm min^−1^ (mean±s.d., *n*=126) as compared with controls with 7.5±2.1 μm min^−1^(*n*=137) (Fig. 3b–d; Supplementary Movie 7). Reconstitution of *forA*^*−*^ cells with GFP-tagged ForAΔDAD restored motility to wild-type levels, demonstrating the absence of ForA to be the sole cause for the phenotype (Fig. 3c). Notably, however, when compressed under a sheet of agar, *forA*^*−*^ cells were strongly impaired in 2D-confined cell migration (3.0±1.0 μm min^−1^, *n*=190) as opposed to wild type (4.3±1.7 μm min^−1^, *n*=128) (Fig. 3b,e,f; Supplementary Movie 8). Thus, when comparing migration in unconfined versus 2D-confined settings, the speed of wild-type cells was reduced by 43%, whereas the speed of *forA*^*−*^ cells was diminished by 73%. Since myosin-II-based contractility is necessary to allow migration in confined environments (see below), these findings pointed towards a defect associated with the contractile machinery at this point.

### IQGAPs and cortexillin localize like myosin II to the rear

**Figure 4:**
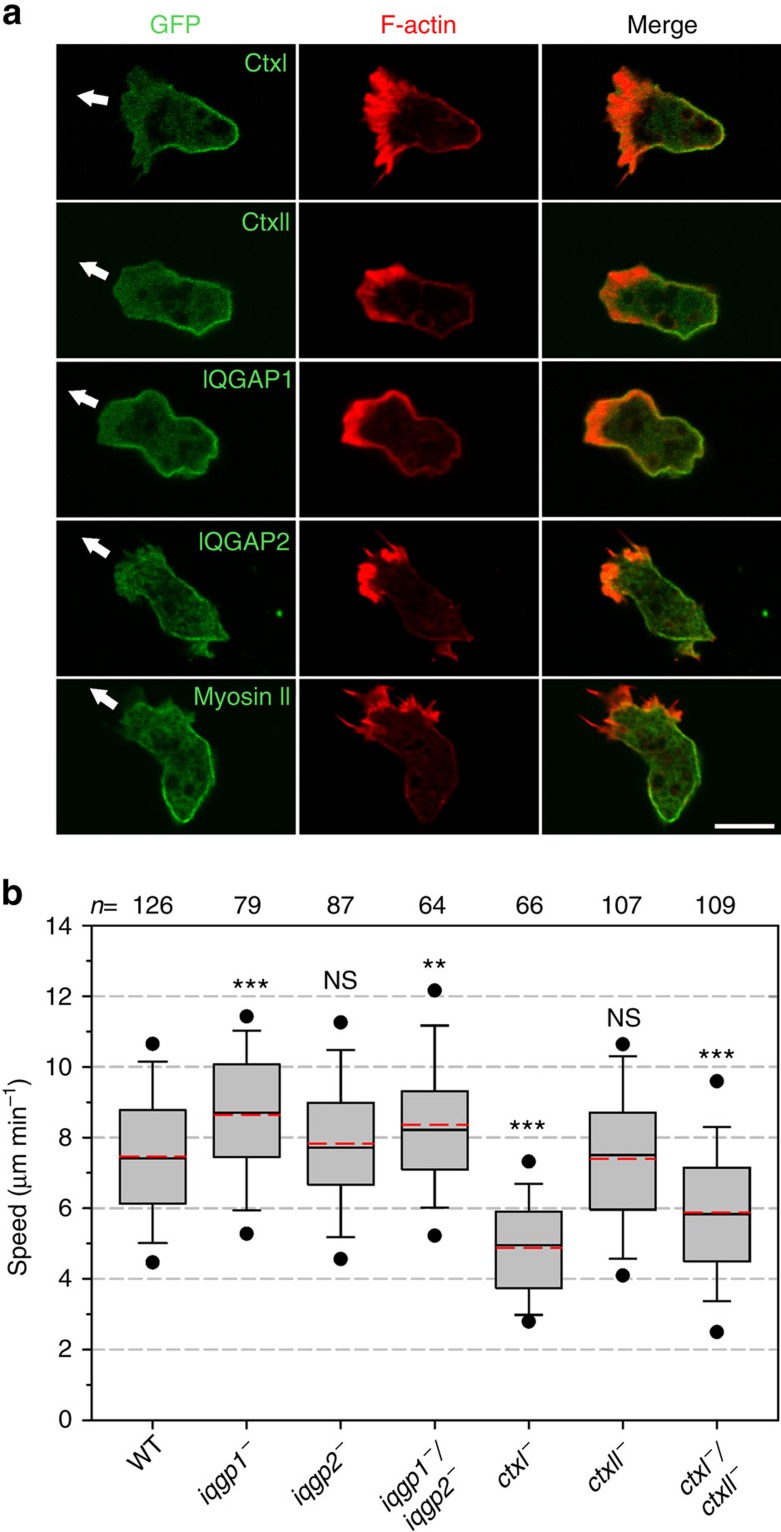
Ctx and the IQGAPs localize like myosin II to the rear and affect cell migration. (**a**) The proteins indicated were expressed as GFP fusions in the respective knockout mutants, fixed and labelled with Atto488-conjugated nanobodies (green) to enhance the GFP signal. Filamentous actin was stained with rhodamine phalloidin (red). The white arrow marks the direction of cell movement. Scale bar, 10 μm. (**b**) Quantitative analysis of random cell motility of *iqgp*^*−*^ and *ctx*^−^ mutants in unconfined environments. *n*, number of tracked cells. ***P≤0.001; **P≤0.01; NS, not significant (Mann–Whitney *U*-test). Statistical differences refer to wild type.

The increased speed of freely migrating *forA*^*−*^ cells was reminiscent of the increased motility rates of a *Dictyostelium* mutant lacking the IQGAP-related protein IQGAP1 (DGAP1)^27^, suggesting a potential mechanistical crosstalk of IQGAP1 and ForA in cell migration. The scaffolding protein IQGAP1 and the closely related IQGAP2 (GAPA) are tightly associated with the heterodimeric actin-bundling protein cortexillin (Ctx), forming preferentially antiparallel bundles *in vitro*^28^, and notably, all of them are also found in the cleavage furrow of dividing cells^29^,^30^,^31^. We therefore analysed the localization of these proteins fused to GFP in reconstituted mutant backgrounds. Similar to the localization of active ForA and myosin II in polarized, migrating growth-phase cells, both IQGAPs as well as the Ctx isoforms I and II were enriched in the rear cell cortex as opposed to the strong accumulation of F-actin in cell fronts (Fig. 4a). Analysis of cell migration in single and double mutants revealed generally increased motility rates for the *iqgp*^*−*^ mutants, whereas motility of *ctx*^−^ cells was diminished as compared with the wild type, although the effects of IQGAP2 and CtxII for cell migration in the single mutants were negligible (Fig. 4b).

### ForA is required for cortical integrity

**Figure 5:**
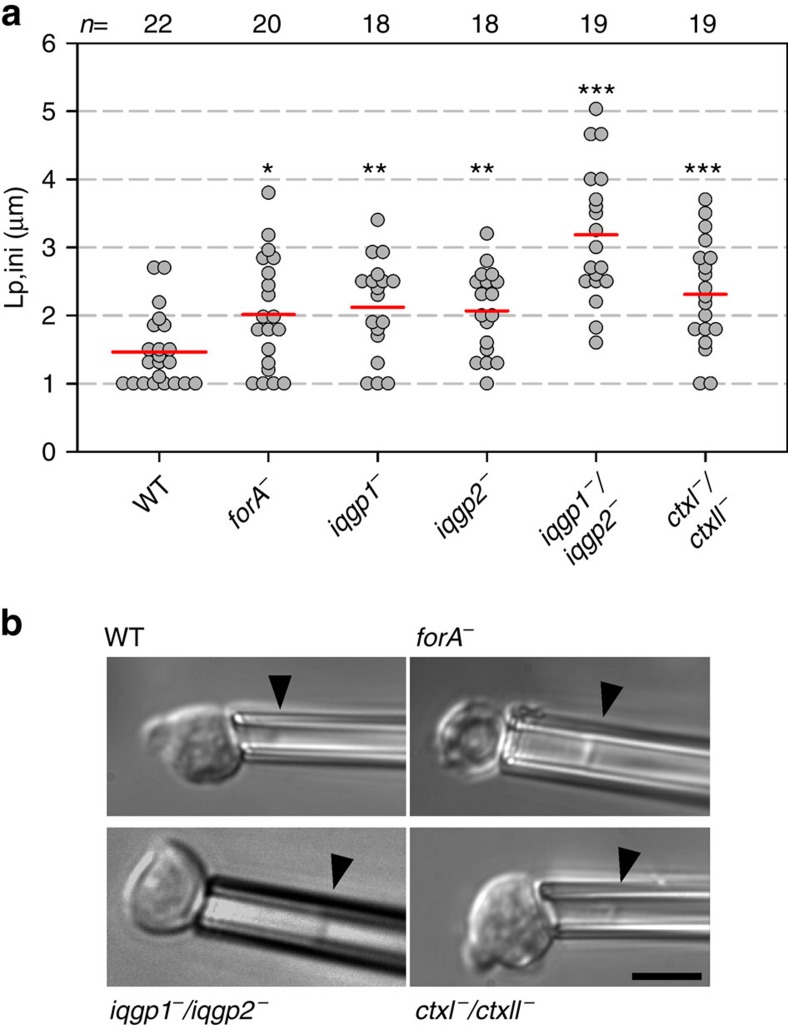
ForA is required for cortical integrity. (**a**) Initial projection length *L*_p,ini_ of wild-type and mutant cells determined by MPA using a constant suction pressure of 500 Pa. Red lines, mean values. *n*, number of analysed cells. ***P≤0.001; **P≤0.01; *P≤0.05 (Mann–Whitney *U*-test). Statistical differences refer to wild type. (**b**) Development of projections (black arrowheads mark menisci) after 145 s in representative samples. Scale bar, 10 μm.

The IQGAPs and Ctx were previously shown to be implicated in regulation of cortical tension in adherent cells^32^,^33^. To assess the role of ForA in cortical integrity in the absence of adhesion forces and compare its contributions with those of the IQGAPs and Ctx in the same experimental set-up, we employed MPA to measure the global mechanical rigidity of freely floating cells, as all of these proteins are evenly distributed in the cortex of unpolarized cells (Fig. 1a; refs 30, 31). To preclude secondary responses of the cells to external suction pressure, we quantified the initial projection length (*L*_p,ini_) of cells captured from suspension at a constant pressure of 500 Pa in MPA assays. Loss of ForA, the IQGAPs or of Ctx resulted in significantly longer projection lengths, indicating more unstable cell cortices as compared with the wild type (Fig. 5a,b). Of note, with time only wild-type cells as well as *forA*^*−*^ and *iqgp1*^*−*^ single mutants were able to counteract the suction pressure and actively withdraw from the micropipette, while 16% of the *ctxI*^*−*^/*ctxII*^−^ and 84% of the *iqgp1*^−^/*iqgp2*^−^ mutants were entirely aspirated (Supplementary Movie 9). This indicated that both double mutants are unable to appropriately reorganize their cortical actin network or suffer from defects in mechanotransduction to counteract the applied force through increased actomyosin-based contractility. We therefore reasoned that the increased motility of *forA*^*−*^ cells as well as of *iqgp1*^−^ and *iqgp1*^−^/*iqgp2*^−^ mutants could be due to a weakened cell cortex allowing enhanced formation of protrusions in the front. The *ctxI*^*−*^ and *ctxI*^*−*^/*ctxII*^−^ mutants though, despite their more fragile cortices, may be slower due to impaired cortex–membrane attachment and filament bundling deteriorating force transmission from the contractile actomyosin system to the plasma membrane at the trailing edge^33^.

### ForA synergizes with myosin II in motility

**Figure 6:**
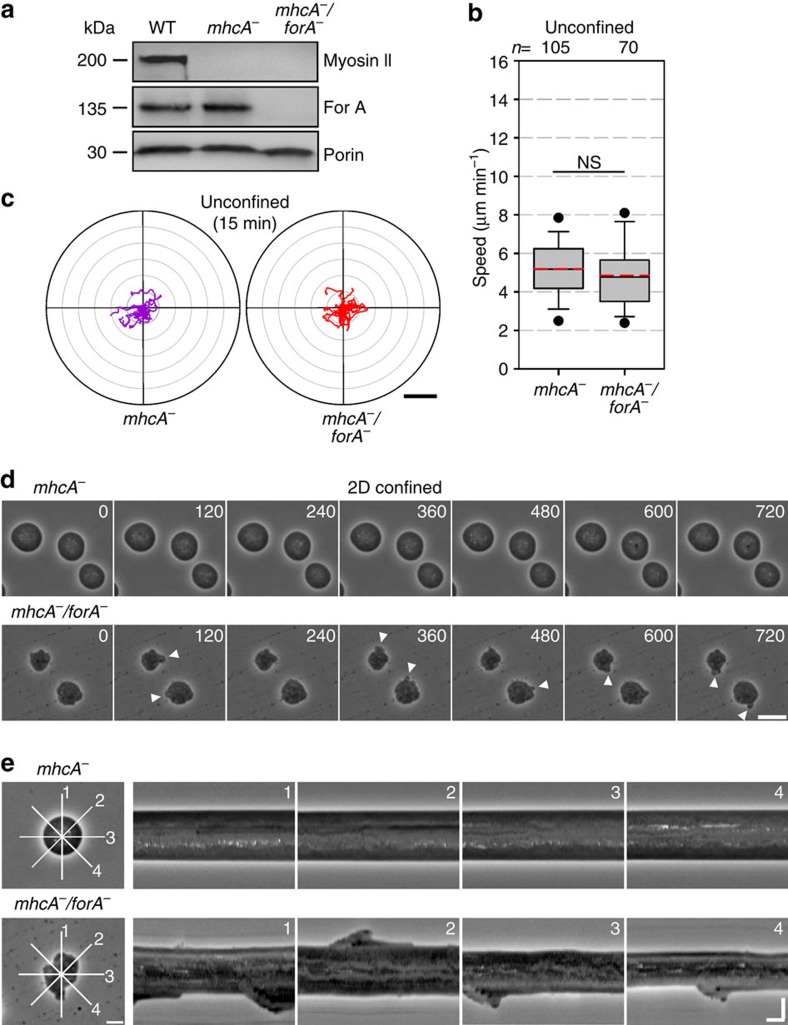
The ForA motility phenotypes are myosin II dependent. (**a**) Elimination of ForA in *mhcA*^*−*^ cells was confirmed by immunoblot using myosin-II-specific antibodies. Porin served as a loading control. (**b**) Quantitative comparison of random cell migration of *mhcA*^*−*^ and *mhcA*^*−*^/*forA*^*−*^ cells in unconfined environments revealed no statistical difference. *n*, number of tracked cells. NS, not significant (Mann–Whitney *U*-test). (**c**) Radar plots of 20 cell trajectories each taken from representative samples. Scale bar, 40 μm. (**d**) Still images from time-lapse phase-contrast movies refer to Supplementary Movie 8 and illustrate that both cell lines are unable to migrate under agar. However, only *mhcA*^*−*^/*forA*^*−*^ cells were able to form protrusion along their periphery (white arrowheads) in 2D confinement. Scale bar, 20 μm. (**e**) Kymograph analyses of *mhcA*^*−*^ and *mhcA*^*−*^/*forA*^*−*^ cells, respectively, that were selected from the movies depicted in **d**. Kymographs were generated along the indicated lines. Horizontal scale bar, 100 s. Vertical scale bar, 10 μm.

To assess the relevance of myosin-II-based contraction in this context and additionally either confirm or exclude a potential competition between different actin nucleators^34^, we disrupted the *forA* gene in myosin-II-null cells (Fig. 6a). Analysis of unconfined random cell migration of *mhcA*^*−*^/*forA*^*−*^-double mutants revealed that loss of ForA no longer enhanced motility in this genetic background (Fig. 6b,c; Supplementary Movie 10), demonstrating that this motility phenotype was strictly dependent on myosin II activity. A quantitative comparison of these mutants in 2D-confined environments was not possible due to the inability of the cells to polarize and migrate under agar in the absence of myosin II (Fig. 6d; Supplementary Movie 11). Despite the fact that *mhcA*^*−*^/*forA*^*−*^-double mutants are immotile, time-lapse imaging and kymograph analyses interestingly revealed that these cells were still able to form small protrusions along their entire periphery, while *mhcA*^*−*^ cells had an entirely spherical and smooth surface illustrating a complete lack of protrusive activity (Fig. 6d,e; Supplementary Movie 11). These observations are therefore not only consistent with the importance of myosin II in symmetry breaking, but additionally support the inhibitory role of ForA in the formation of cell protrusions, as the protein is evenly distributed in the cell cortex of unpolarized cells (see Fig. 1a; Supplementary Movie 11). Combined, they further demonstrate that under 2D confinement, hydrostatic pressure generated by actomyosin-based contractility must synergize with protrusive activities in the front to drive cell migration.

### ForA is required to withstand high actomyosin-based forces

**Figure 7:**
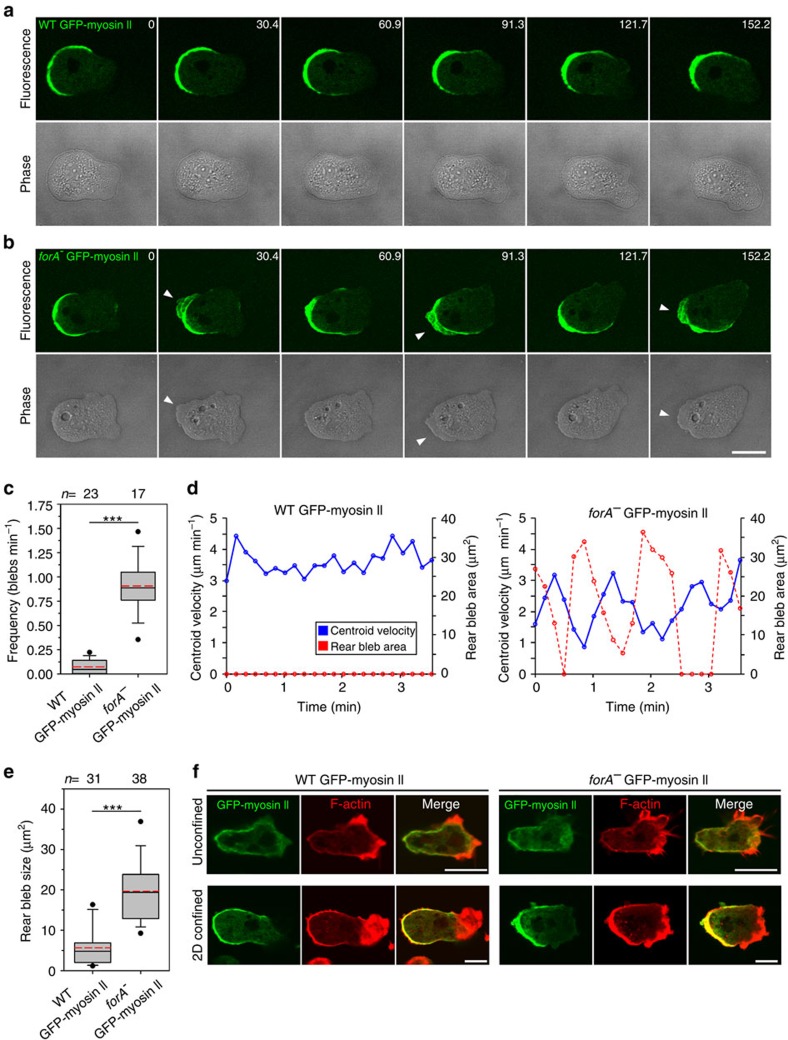
ForA prevents blebbing at the rear during cell migration in 2D-confined environments. (**a**,**b**) Accumulation of GFP-myosin II in the rear of wild-type and *forA*^*−*^ cells migrating under agar. Arrowheads indicate posterior blebs formed in *forA*^*−*^ cells. Images refer to Supplementary Movies 12 and 13. Scale bar, 10 μm. (**c**,**e**) Frequency and size of posterior blebs in wild-type and *forA*^*−*^ cells expressing GFP-myosin II under agar. *n*, number of analysed blebs. ***P≤0.001 (Mann–Whitney *U*-test). (**d**) Inverse correlation of cell motility and rear blebbing. Centroid velocities (blue solid line) and bleb areas (red dashed line) of representative frames shown in **a** and **b**. Excessive blebbing of *forA*^*−*^ cells resulted in diminished migration speed. (**f**) Increased accumulation of myosin II and filamentous actin in the rear after compression under agar. After fixing the cells, GFP-tagged myosin II was enhanced with Atto488-conjugated nanobodies (green). Filamentous actin was stained with rhodamine phalloidin (red). Scale bars, 5 μm.

Exposure of *Dictyostelium* cells to mechanical stress as seen in agar overlay assays is associated with increased myosin II enrichment at the cortex, leading to an augmentation of contraction to counteract the compressive pressure^35^. To directly visualize the contractile machinery in 2D-confined environments, we expressed GFP-tagged myosin II in wild-type and *forA*^*−*^ cells and analysed their migration by time-lapse confocal imaging under agar. GFP-myosin II was strongly enriched at the rear cortex of migrating wild-type-derived cells (Fig. 7a; Supplementary Movie 12). GFP-myosin II localization in *forA*^*−*^-derived cells was similar, however, and as opposed to control, time-lapse imaging interestingly revealed that these cells repeatedly formed extensive blebs in their rear (Fig. 7b; Supplementary Movie 13). Blebs form due to detachment of the plasma membrane from a weakened or ruptured cortex by hydrostatic pressure generated by myosin-II-driven contraction^36^. Quantitative analyses showed a 13-fold higher occurrence (0.91±0.27 s^−1^, mean±s.d., *n*=17 versus 0.07±0.07 s^−1^, *n*=23) and an about a fourfold larger size (19.66±7.89 μm^2^, mean±s.d., *n*=38 versus 5.66±4.66 μm^2^, *n*=31) of these rearward blebs in *forA*^*−*^ cells as compared with control (Fig. 7c,e). Strikingly, the determination of centroid velocity and rear bleb size revealed a periodic and inverse correlation between cell speed and blebbing activity in the mutant, as periods with bleb growth accurately matched phases with decreased cell speed (Fig. 7d; Supplementary Movie 14). ForA is obviously not required for myosin-II-driven contraction *per se*, as evidenced by the excessive blebbing in GFP-myosin-II-expressing *forA*^*−*^ cells, but is a critical component to prevent posterior blebbing. Fluorescence labelling of fixed cells further revealed a substantially increased accumulation of F-actin in the rear of compressed cells as opposed to freely moving cells (Fig. 7f). Hence, in confined environments the cortex is strengthened by relocation of actin, myosin II and other actin-binding proteins such as the Ctx/IQGAP1 complex (Supplementary Fig. 3) to the rear. However, in spite of these adaptations the posterior cortical actin meshwork in the formin mutant was evidently still not able to provide the mechanical strength of the cortex required for efficient cell migration in 2D-confined environments. Thus, ForA serves to protect the cell against unproductive blebbing upon the increased contractile forces in response to mechanical stress by generating a specialized subset of actin filaments as the foundation of the protective actin sheath in the rear cortex to drive motility.

### Recruitment of ForA to the cell rear

**Figure 8:**
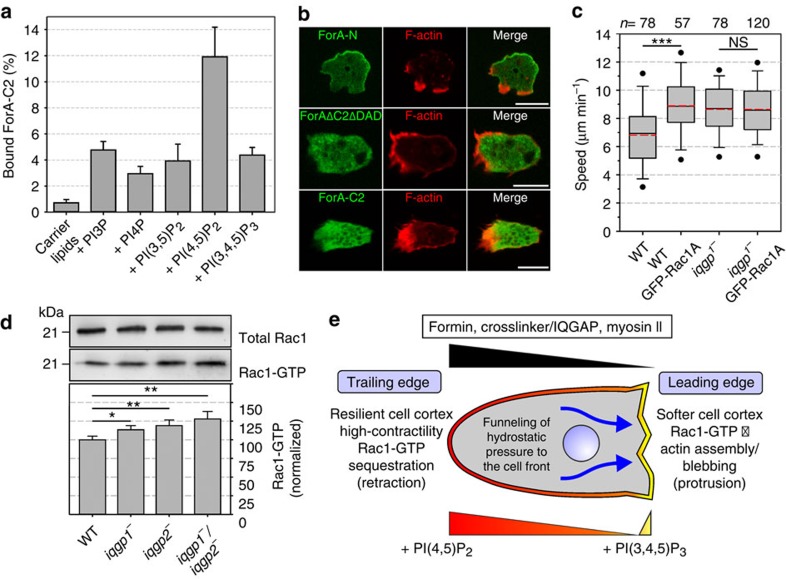
Regulatory mechanisms establishing cortical rigidity in the rear. (**a**) The ForA-C2 domain binds preferentially to PI(4,5)P_2_ *in vitro*. Quantification of ForA-C2 binding to phosphoinositide-containing liposomes. Data are mean±s.d., *n*=4. (**b**) ForA-C2 is essential for subcellular targeting. After reconstitution in *forA*^*−*^ cells with truncated ForA constructs fused to GFP, the cells were fixed and labelled with Alexa488-conjugated nanobodies (green) and rhodamine phalloidin to visualize filamentous actin (red). Deletion of C2 abolished trailing edge localization, but C2 alone was not sufficient for targeting to the rear. Scale bar, 10 μm. (**c**) Ectopic expression of GFP-tagged Rac1A increases random motility in wild-type cells, but not in *iqgp1*^−^ cells. *n*, number of analysed cells. ***P≤0.001; NS, not significant (Mann–Whitney *U*-test). (**d**) MBP-PAK1-GBD pull-downs showed increased levels of active Rac1 in single- and double-*iqgp*^*−*^ mutants. Error bars represent mean±s.d., *n*=4. *P≤0.05; **P≤0.01 (two-tailed, unpaired Student's *t*-test). (**e**) Model PI(3,4,5)P_3_ signalling guides protrusion to the front^10^, whereas PI(4,5)P_2_ positions the resilient cortical meshwork together with contraction machinery to the rear. For more details see text.

Next, we asked how ForA is positioned in the trailing edge of cells. The protein contains a putative C2 domain at its N terminus (aa 1–100), suggesting electrostatic interactions with acidic phospholipids^37^. To directly test interaction of this region with lipids *in vitro*, purified GST-tagged ForA-C2 was assessed by co-flotation assays with liposomes containing various phosphoinositides. ForA-C2 bound preferentially to phosphatidyl-inositol-(4,5)-bisphosphate (PI(4,5)P_2_) (Fig. 8a), which accumulates in the trailing edge of migrating cells^10^. To directly test whether the C2 domain is critical for targeting *in vivo*, we analysed the localization of the N-terminal half of ForA containing the C2 domain (ForA-N), the active formin lacking C2 (ForAΔC2ΔDAD) as well as the C2 domain alone (ForA-C2) in *forA*^*−*^ cells. While GFP-tagged ForA-N localized to the cortex in the rear like the active formin, both ForAΔC2ΔDAD and ForA-C2 were evenly distributed in the entire cytoplasm (Fig. 8b), demonstrating that ForA-C2 interaction with PI(4,5)P_2_ is essential but not sufficient for targeting to the cell rear. Thus, additional signals seem to be mandatory for subcellular localization. Obviously, ForA targeting does not require actin, since the appropriately localizing ForA-N lacks all actin interaction surfaces. Since CtxI was previously also shown to interact with PI(4,5)P_2_ (ref. 38), the Ctx/IQGAP complex may be recruited in a similar fashion and mediate the crosslinking of ForA-generated actin filaments beneath the plasma membrane.

### IQGAPs sequester active Rac1 in the rear

We then addressed the somewhat unexpected localization of the Rac1-binding protein IQGAP1 in the rear^39^, because Rac1 is well established and mandatory to induce migratory protrusions downstream of Scar/WAVE signalling in Arp2/3 complex-mediated actin assembly in the front^40^. Consistently, overexpression of wild-type Rac1 leads to increased Rac1-GTP levels and augments motility of *Dictyostelium* cells^27^. We noticed, however, that wild-type Rac1 overexpressed at similar levels in the faster *iqgp1*^−^ mutants did not further increase cell speed (Fig. 8c; Supplementary Fig. 4). Since both IQGAPs interact exclusively with active Rac1 (refs 30, 41), we reasoned that presumably due to elevated levels of accessible Rac1-GTP caused by absence of the Rac1 effector IQGAP1, the Arp2/3 complex might be already fully activated in *iqgp1*^−^ mutants to drive actin assembly in the leading edge. In line with this, the fast *iqgp1*^−^ mutants were previously reported to generate excessive cell protrusions, while the overexpression of IQGAP1 led to round cells largely devoid of pseudopods and filopodia^42^. To test our hypothesis, we performed GTPase activation assays using maltose-binding protein (MBP)-tagged PAK1-GBD and indeed found higher levels of active Rac1 in the *iqgp*^*−*^ mutants (Fig. 8d). Thus, in addition to their effects on cortical integrity, the IQGAPs seem to suppress activation of residual amounts of Arp2/3 complex in the rear by sequestration of active Rac1.

### ForA is the functional homologue of vertebrate mDia1

**Figure 9:**
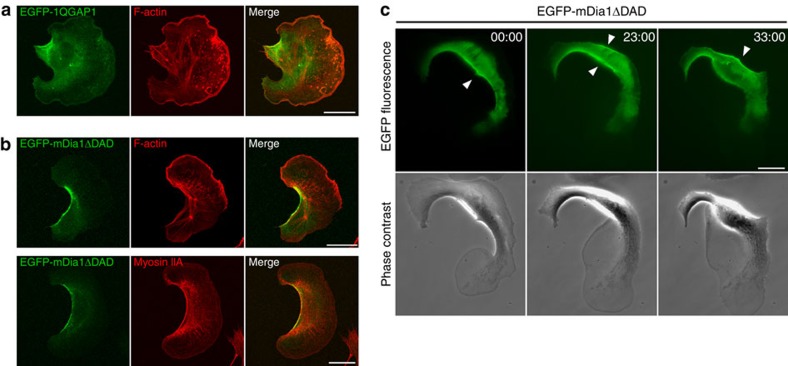
Subcellular localization of IQGAP1 and active mDia1 in B16-F1 cells. (**a**) B16-F1 melanoma cells, ectopically expressing EGFP-tagged IQGAP1, were plated on laminin-coated glass coverslips, fixed and labelled with Alexa488-conjugated nanobodies (green) and rhodamine phalloidin to visualize filamentous actin (red). Note the enrichment of IQGAP1 in the rear. (**b**) Subcellular localization of ectopically expressed EGFP-tagged active mDia1 in B16-F1 cells migrating on laminin. The cells were processed as described in **a**, but were additionally stained for myosin IIA. Active mDia1 was excluded from the leading edge and other actin-rich structures, with the exception of its prominent cortex localization in the trailing edge, where it colocalized together with cortical F-actin and myosin IIA. (**c**) mDia1 dynamics in repolarizing cells. Relocalization of active mDia1 in repolarizing B16-F1 cells to the new prospective end is indicated by white arrowheads and comparable to active ForA in *Dictyostelium* cells. Still images from time-lapse movies are shown. Time corresponds to min:s. Scale bars, 20 μm.

Since actomyosin-based contraction is exploited by many cell types to drive or support motility, we finally asked whether higher eukaryotes could employ a related mechanism to generate a rigid cortex in the rear. Although vertebrates do not express Ctx, human IQGAP1 by itself might replace its function, since it was previously reported to crosslink actin filaments in *vitro*^43^, and have shown to accumulate in the trailing edge, at least in B16-F10 and WM239A melanoma cells^44^,^45^. To corroborate these findings in a commonly used and highly migratory cell line, we transfected B16-F1 mouse melanoma cells with enhanced (E)GFP-tagged IQGAP1 (ref. 46) and monitored its subcellular localization. In moderately expressing cells IQGAP1 localized rather weakly to actin-based structures in the front, but was substantially enriched in the trailing edge (Fig. 9a). Since contractility in vertebrates is regulated by Rho signalling to mDia1 and ROCK to trigger actin polymerization and activate myosin^47^, we additionally monitored localization of constitutively active EGFP-tagged mDia1 (EGFP-mDia1ΔDAD). Strong overexpression of active mDia1 in B16-F1 cells induced aberrant cell morphologies and led to massive disorganization of the F-actin cytoskeleton. By contrast, in moderately expressing cells, active mDia1 was excluded from the leading edge, but profoundly localized like ForA to the cortex of the trailing edge together with cortical F-actin and myosin IIA (Fig. 9b; Supplementary Movie 15). Active mDia1 also relocated to the new prospective rear in repolarizing cells and vanished before the formation of migratory protrusions (Fig. 9c). These findings collectively suggest that *Dictyostelium* ForA represents the functional homologue of mDia1 pointing towards a conserved mechanism in the generation of the rear cortex across species.

## Discussion

Amoeboid cell migration is context dependent and governed by the balanced interplay of cell–substrate adhesion, together with the cortical activities eliciting protrusion and contraction^5^. The results reported here provide new insights into the molecular mechanisms involved in cortex formation, mechanics and its function during migration of *Dictyostelium* cells in unconfined and 2D-confined environments. On the basis of the data, we propose that ForA initiates the formation of linear filaments beneath the plasma membrane that are mechanically reinforced by the antiparallel actin crosslinker Ctx and IQGAP-related proteins to generate a specialized actin meshwork in the rear. This resilient compartment is necessary to withstand the amplified actomyosin-based contractile forces in 2D-confined environments and to drive motility. Our hypothesis, diagrammed in Fig. 8e, suggests that in wild-type cells internal hydrostatic pressure generated by myosin-II-based contraction is efficiently gated by the robust cortex in the rear to the softer front to promote cell protrusion. Consistently, similar to *ctx*^−^ and *iqgp*^*−*^ mutants, *forA*^*−*^ cells show substantial defects in cortical rigidity as evidenced by the aspiration assays. Moreover, the impaired migration of *forA*^*−*^ cells under agar was associated with excessive blebbing in regions with highest myosin II accumulation. This illustrates not only the mechanical weakness of the rear cortex but also the ability of the cells to contract in the absence of the formin in spite of a generally increased enrichment of actin, myosin II, Ctx and IQGAP to the back (Fig. 7f; Supplementary Fig. 3). These findings therefore clearly argue against instantaneous pressure equilibration in the cytosol and instead support the concept of non-equilibration of heterogeneous transients in the pressure field of blebbing cells^48^. Interestingly, the migration of MDA-MB-231 human breast adenocarcinoma cells in three-dimensional Matrigel was previously reported to be exclusively driven by blebbing in the trailing edge^49^. How can these apparently contradictory findings be reconciled? We hypothesize that in three-dimensional Matrigel, rear blebs of MDA-MB-321 cells could provide repulsive forces in the rear by pushing against the adjacent collagen fibres of the matrix to move the cells forward, while in *Dictyostelium* even the extensive posterior blebs formed under agar cannot exert repulsive forces against the surrounding liquid due to its low mechanical resistance.

Myosin-II-null cells are motile in unconfined conditions, albeit they migrate with only half the speed of wild-type cells^24^. Subsequent work revealed two mechanically distinct modes of amoeboid movement characterized by the formation of two different cell-surface protrusions, namely actin-rich pseudopods or blebs^50^. The hydrostatic pressure-driven blebs are strongly impaired by perturbation of myosin-II-based contractility or changes in external osmolarity that affects internal hydrostatic pressure. Our analyses of mutants lacking myosin II and ForA in different combinations allowed us to expand on these findings and assess the interplay and contribution of these proteins for motility in unconfined and 2D-confined scenarios. Intriguingly, we observed an almost doubled migration speed of *forA*^*−*^ cells as compared with wild-type cells in unrestricted conditions. This enhanced motility phenotype is dependent on myosin II function (Fig. 6b,c), demonstrating involvement of actomyosin contraction associated with generation of hydrostatic pressure in this process. Given the less rigid cortical actin meshwork in *forA*^*−*^ cells and provided that the strength of actomyosin-generated forces and the resulting pressure in wild-type and *forA*^*−*^ cells are comparable, we assume that the increased speed of the mutant is caused by increased compliance of the cortex allowing easier compression of the cells in the back and easier deformability in their front. In 2D confinement, however, augmented hydrostatic pressure evidently exceeds a tolerable limit that disrupts the rear cortex leading to unproductive blebbing that diminishes motility of these cells. Moreover, we found that *mhcA*^*−*^ cells are unable to migrate under agar (Fig. 6d), which emphasizes the importance of myosin-II-based contractile forces and generation of hydrostatic pressure for motility of *Dictyostelium* cells in 2D confinement. Although the *mhcA*^*−*^/*forA*^*−*^ cells were also immotile under agar, in contrast to *mhcA*^*−*^ cells, they still formed small protrusions at their periphery. We therefore conclude that in 2D confinement, hydrostatic pressure generated in the rear must synergize with protrusive activities in the leading edge to allow cell migration.

Efficient cell migration not only requires protrusion in the front and contraction in the back, but also entails inhibition of lateral and rear protrusions. This function appears also to be ascertained by the ForA-based cortical meshwork as we were unable to detect the formation of any protrusion from cortical regions with high ForA accumulation. In case protrusions were spontaneously formed in the rear, these events were invariably preceded by disappearance of the formin (Fig. 1b). This notion is consistent with the lack of protrusions in unpolarized *mhcA*^*−*^ cells under agar, which still contain ForA evenly distributed in their cell cortex, and was further substantiated by the dynamic redistribution of ForA in repolarizing cells (Fig. 1c,d). Consistent with our data and previous work^51^, we further propose that inhibition of lateral protrusion in migrating cells is additionally sustained by the IQGAPs through sequestration of active Rac1 to prevent undesirable activation of the Arp2/3 complex.

In vertebrates, cortical actin flow and myosin II have been proposed to localize components of the contractile machinery to the rear^52^,^53^. However, in migrating *Dictyostelium* cells, F-actin was reported to move rearward with a much slower velocity than the movement of the centroid^54^. Thus, it is unlikely that actin flow directly controls cell migration in these cells. The distribution of ForA is also myosin II independent, as the formin is still actively recruited to the rear in myosin-II-null cells (Supplementary Fig. 5). We instead found that ForA localization is regulated by PI(4,5)P_2_ that is enriched in the trailing edge of polarized cells^10^. Thus, the previously reported defect of mutants lacking PTEN (phosphatase tensin homologue)—which is the main enzyme converting phosphatidyl-inositol-(3,4,5)-triphosphate (PI(3,4,5)P_3_) back to PI(4,5)P_2_—in the inhibition of lateral protrusions may be caused by the inability of ForA to localize in the absence of a perturbed PI(4,5)P_2_ differential^55^. By the use of truncated ForA constructs, we show that the PI(4,5)P_2_-binding C2 domain is essential, although alone, it is not sufficient for targeting to the rear. Since the accurately targeting N-terminal fragment not only encompasses the C2 domain, but also contains a GTPase-binding site, targeting to the rear most likely requires the concurrent interaction of both binding sites with PI(4,5)P_2_ and a as yet unknown activated GTPase. Consistently, mDia1 lacking either its PI(4,5)P_2_-interacting N-terminal basic region or its GBD fail to localize to the membrane^56^,^57^.

Finally, we provide evidence that related mechanisms could also be utilized by higher eukaryotes. Although vertebrates do not express Ctx, we propose that human IQGAP1 might already substitute its function. Consistent with previous work^45^,^58^, we show that IQGAP1 accumulates in the trailing edge of B16-F1 cells (Fig. 9a). Our hypothesis is further supported by the ability of IQGAP1 to crosslink actin filaments, as well as the presence of a PI(4,5)P_2_-binding site as found in CtxI^43^,^44^,^38^. It is quite possible, however, that in vertebrates crosslinking and membrane attachment of cortical actin filament is additionally supported by other crosslinkers, for instance the PI(4,5)P_2_-activated ERM proteins^59^. Importantly, for the first time, we show localization of active mDia1 in the rear cortex of migrating cells. mDia1 colocalized together with cortical F-actin and myosin IIA, thus suggesting a critical role in rear cortex function (Fig. 8b). This is consistent with the blebbing phenotype of mDia1-depleted cells and enrichment of mDia1 at the cell rear of invading cells during entosis^23^,^60^. Moreover, mDia1-depleted cells were previously shown to be impaired in polarization, migration and chemotaxis^61^. mDia1 shares several main features with ForA, suggesting that this DRF and ForA represent functional homologues: (i) it is localized to the rear of migrating cells; (ii) the interaction of N-terminal residues with PI(4,5)P_2_ is required but not sufficient for membrane targeting and it additionally requires RhoA signalling; (iii) it dynamically altered its position in turning B16-F1 cells (Fig. 9c). Despite the fact that mDia1 is one of the best characterized formins in the field, it is rather surprising why its prominent rear localization in migrating cells has not been discovered previously. We assume that this may be due to the use of different cell types, overexpression or transfections with N-terminally truncated constructs lacking PI(4,5)P_2_ or the GTPase-interaction surfaces.

## Methods

### Plasmids

For expression of ForA GFP fusion proteins, first ForA-N (aa 1–647) was amplified by PCR as a BamHI/SalI fragment using complementary DNA from AX2 wild-type cells as template (European Nucleotide Archive accession number LN870236), and cloned into the BamHI/SalI sites of pDGFP-MCS-Neo^27^. FL ForA (aa 1–1,218) was generated by insertion of the remaining coding sequence of the *forA* gene into the SpeI/SalI sites of GFP-ForA-N. GFP-ForAΔDAD (aa 1–1,138) and GFP-ForAΔC2ΔDAD (aa 91–1,138) were obtained by PCR using ForA-FL as a template, the fragments were digested with BamHI/SalI and inserted into the corresponding sites of pDGFP-MCS-Neo. For expression of GFP-tagged C2, a fragment corresponding to residues 1–99 was amplified by PCR and inserted into pDGFP-MCS-Neo. For expression of ForA constructs in *Escherichia coli* ForA-FH3 (aa 139–539), ForA-FH3L (aa 91–647), ForA-C2 (aa 1–99) and ForA-C (aa 635–1,218) were amplified by PCR and inserted into the BamHI and SalI sites of pGEX-6P-1 (GE Healthcare). For expression of MBP-GBD-PAK1, the coding sequences corresponding to residues 57–200 of rat PAK1 complementary DNA were amplified by PCR and inserted into the BamHI/SalI sites of pMAL-c2X (NEB). The plasmids for expression of GST- and GFP-tagged Rac1A have been reported^42^. The *forA* knockout vector was constructed by amplification of a 750-base pair 5′ PstI/BamHI fragment and a 900-base pair 3′ SalI/HindIII fragment of the *forA* gene from genomic DNA, respectively, and insertion of the fragments into the corresponding sites of pLPBLP^62^. Primer sequences of all constructs are provided in Supplementary Table 1. The sequences of all constructs were verified by sequencing. The plasmid for expression of GFP-tagged myosin-II heavy chain was pBigGFPmyo^63^. For transfection of B16-F1 mouse melanoma cells, mDia1ΔDAD^56^ was used. EGFP-tagged IQGAP1 was provided by Addgene (Plasmid #30112).

### Cell culture and transfections

*D. discoideum* cells were grown in HL5-C medium including glucose (Formedium) and were transformed by electroporation with an Xcell gene pulser (Bio-Rad) using pre-set protocol 4–6 for *Dictyostelium*. The *forA*^*−*^ and *mhcA*^*−*^*/forA*^*−*^ cells were obtained by disruption of the *forA* gene in AX2 wild-type cells and in the *mhcA*^*−*^ mutant^24^, respectively, by homologous recombination using the pLPBLP-based targeting vector system^62^ after digestion of the targeting vector with BamHI and SalI. Stably transfected cells were selected with 10 μg ml^−1^ blasticidin S (Invivogen). Gene disruption was initially screened by PCR and eventually confirmed by the absence of the protein in immunoblots. Cell lines expressing GFP fusion proteins were obtained by transfecting the cells with the appropriate plasmids and selecting the transformants with 10 μg ml^−1^ of geneticin (Sigma). B16-F1 mouse melanoma cells (ATTC CRL-6323) were grown in high-glucose DMEM (Biowest) supplemented with 10% FCS (Biowest) and 2 mM glutamine (Sigma). Transfections were performed overnight using JetPrime (Polyplus) according to manufacturers' instructions.

### Protein purification

Expression of GST- or MBP-tagged proteins was induced in *E. coli* strain Rossetta 2 (Novagen) with 1 mM isopropyl-b-D-thiogalactoside at 21 °C for 12 h. The proteins were subsequently purified from bacterial extracts by affinity chromatography using either glutathione-conjugated agarose (Sigma-Aldrich) or amylose high-flow resin (NEB) followed by size-exclusion chromatography on an Äkta Purifier System equipped with a HiLoad 26/600 Superdex 200 column (GE Healthcare). In case of GST-ForA-C, before size-exclusion chromatography, the GST-tag was cleaved by PreScission protease (GE Healthcare), and uncleaved GST-ForA-C and GST were removed by a second affinity chromatography using glutathione-conjugated agarose. The purification of heterodimeric capping protein (CP)^56^, mDia1-C^56^ and *Dictyostelium* PFN I was performed accordingly. The purified proteins were dialysed against storage buffer (100 mM KCl, 1 mM dithiothreitol (DTT), 60% glycerol and 20 mM HEPES pH 7.4) and stored at −20 °C for later use. Actin was purified from acetone powder of rabbit skeletal muscle according to standard procedures and labelled on Cys374 with ATTO 488 maleimide (ATTO-TEC) or with *N*-(1-Pyrenyl)maleimide (Invitrogen), respectively.

### Actin biochemistry

The polymerization of 2 μM rabbit skeletal muscle G-actin (5% pyrene labelled) was initiated with 1 × KMEI (50 mM KCl, 1 mM MgCl_2_, 1 mM EGTA and 10 mM imidazol, pH 7.0) and was monitored in 96-well plates either alone or in the presence of various concentrations of ForA-C using a Synergy 4 fluorescence microplate reader (Biotek). To quantify formin autoinhibition, 50 nM of ForA-C were incubated with various concentrations of GST-tagged ForA-N in 1 × KMEI for 2 min. Subsequently, 2 μM G-actin were added and the polymerization rates were recorded as above. The maximum slopes of the polymerization curves were normalized, averaged for each data set and plotted against the molar ratio of ForA-N/ForA-C. As 50 nM ForA-C was within the range during which the polymerization rate increased linearly to the concentration of ForA-C and the slopes indicated high-affinity binding of ForA-N to ForA-C, the data were fitted by linear regression. The intersection of the lines marks the molar ratio required for complete inhibition.

For assaying dilution-induced depolymerization experiments, G-actin (50% pyrene labelled) was first polymerized in 1 × KMEI for 2 h at room temperature. Subsequently, the F-actin was preincubated with various concentrations of ForA-C or CP. The depolymerization reaction was initiated by diluting F-actin to 0.1 μM by addition 1 × KMEI buffer into the wells using the automated dispenser of the Synergy 4 plate reader and analysed as above. For *in vitro* TIRF microscopy, ForA-C (10 or 100 nM final concentration) and *Dictyostelium* PFN I (5 μM final concentration) were pre-diluted in 1 × TIRF buffer (20 mM imidazole pH 7.4, 50 mM KCl, 1 mM MgCl_2_, 1 mM EGTA, 20 mM β-mercaptoethanol, 0.5 mM ATP, 15 mM glucose, 2.5 mg ml^−1^ methylcellulose (4,000 cP), 20 μg ml^−1^ catalase and 100 μg ml^−1^ glucose oxidase). The assays were initiated by adding G-actin (1 μM final concentration, 10% Atto488 labelled) and flushing the mixtures into mPEG-silane MW 2000 (Laysan Bio) pre-coated flow chambers. Images were captured with a Nikon Eclipse TI-E inverted microscope equipped with a TIRF Apo × 60 or × 100 objective at 1-s intervals with exposure times of 40 ms by a Ixon3 897 EMCCD camera (Andor) for at least 10 min. The pixel size corresponded to 0.159 μm for the × 100 objective and 0.27 μm for the × 60 objective. The nucleation efficacies were obtained by counting and averaging the number of actin filaments in an area of 80 × 80 μm after 180 s in three independent experiments (*n*=3). The elongation rates of filaments were determined by manual tracking of growing barbed ends using ImageJ software. At least 20 filaments from three movies per condition were measured for the experiments in presence of PFN I.

### Antibodies

Polyclonal antibodies against ForA and Rac1A were raised by immunizing a female New Zealand white rabbit with either recombinant GST-tagged ForA-FH3L or GST-tagged Rac1A following standard protocols. Subsequently, the polyclonal antibodies were affinity purified after coupling of the same antigens to CNBr-activated Sepharose (GE Healthcare). Immunoblotting was performed according to standard protocols using undiluted hybridoma supernatants of IQGAP1-specific mAb 216-394-1 (ref. 30, GFP-specific mAb 264-449-2 (ref. 30), myosin-II-specific mAb 56-396-5 (ref. 64), Porin-specific mAb 70-100-1 (ref. 65) or polyclonal anti-Rac1A and anti-ForA antibodies (1:250 dilution). Alexa488-conjugated nanobodies were from Chromotek (1:200 dilution; #gba488) and polyclonal anti-myosin IIA antibodies were from Thermo Scientific (1:100 dilution; #PA5-17025). Primary antibodies in immunoblots were visualized with phosphatase-coupled anti-mouse (1:1,000, #115-055-62; Dianova) or anti-rabbit IgG (1:1,000; #111-055-046; Dianova). Uncropped scans of the most important immunoblots are shown in Supplementary Fig. 6. For immunohistochemistry, secondary Alexa-555-conjugated goat-anti-rabbit polyclonal antibodies (1:1,000 dilution; #A21429; Invitrogen) were used.

### Fluorescence microscopy and imaging

For immunofluorescence labelling of *Dictyostelium* cells, growth-phase cells expressing GFP-tagged proteins were washed twice with PB, and allowed to adhere on glass coverslips for 20 min. The cells were then fixed with picric acid/paraformaldehyde, permebealized with 70% ethanol, washed extensively with PBS containing 2.7 mM KCl, 1.8 mM KH_2_PO_4_, 10 mM Na_2_HPO_4_, 140 mM NaCl, pH 7.3 and labelled for F-actin with TRITC-conjugated phalloidin (1:200 dilution, #P1951; Sigma). The GFP signal was enhanced by Alexa488-conjugated nanobodies. Fixation of cells compressed under agar was performed accordingly. For live cell imaging, growth-phase cells expressing GFP-tagged proteins were seeded onto 3-cm-diameter glass-bottom dishes (Matek) and allowed to adhere on the glass surface for 20 min. The cells were then washed four times with PB and imaged using an LSM510Meta confocal microscope (Zeiss) equipped with a × 63/1.3 Plan-Neofluar objective using the 488 nm and 543 nm laser lines.

For indirect immunofluorescence microscopy, B16-F1 cells were plated on acid-washed coverslips coated with μg ml^−1^ laminin (Sigma). After fixation with 4% paraformaldehyde in PBS for 15 min, the specimens were permebealized with PBS containing 100 mM glycine and 0.1% Triton X-100 for 5 min. After extensive washing with PBS containing 0.05% (v/v) cold fish gelatine (Sigma) and 0.5% (w/v) bovine serum albumin (Sigma), the cells were stained with the probes indicated. For live cell imaging, the transfected B16-F1 cells were plated onto 3-cm-diameter glass-bottom dishes (Matek) coated with 25 μg ml^−1^ laminin (Sigma), mounted in a heated miniature incubator (Bioscience Tools) and observed at 37 °C with an Olympus IX-81 inverted microscope equipped with a × 40/0.75 numerical aperture UplanFL objective and a cooled CCD camera (CoolSNAP EZ, Photometrics) with a filter wheel and shutters controlled by Metamorph software (Molecular Devices). Data were processed using ImageJ, Adobe Photoshop CS (Adobe Systems) and CorelDraw software (Corel Corporation).

### Analyses of cell migration

Quantitative analysis of random cell motility was performed as follows: growth-phase cells in PB buffer were monitored every 10 s for 15 min by time-lapse imaging at an inverted Olympus IX-81 equipped with × 10 phase-contrast optics (Olympus) and a CoolSnap EZ camera (Photometrics). Migration assays in 2D-confined environments were performed with growth-phase cells that were overlaid with a 0.17-mm-thin sheet of agar (2% in PB buffer). The cells were subsequently allowed to adapt for 90 min to the compression before imaging, which was expanded to 30 min in the motility assays. Single-cell tracks were obtained from recordings with the Track Objects plugin of Metamorph 7 software (Molecular Devices). Data samples of each cell line contain at least 60 individual cells from at least three different experiments. These data were further processed to obtain average speed of single cells in a custom-built protocol operated in Excel (Microsoft). Average speed distributions of analysed samples are presented in box plot representations using SigmaPlot 11.2 (Systat Software Inc). Boxes indicate 25–75 percentiles, the whiskers mark the 10th and 90th, and the outliers the 5th and 95th percentile. Red dashed lines indicate mean values. Centroid velocities were determined from pseudo-phase-contrast time-lapse recordings using the ImageJ plugin BOA of the QuimP11b plugin suite^66^. The instantaneous centroid velocity to a given time point *t* was approximated by calculation of simple moving averages (SMAs) comprising velocities of the previous and following time point amounting to velocity_t,SMA_=(velocity_t−1_+velocity_t_+velocity_t+1_)/3. The size of the posterior blebs was determined using the selection and measurement tools in ImageJ software.

*Analyses of cortical ForA distribution in migrating and repolarizing cells*. Protrusion and retraction velocities as well as cortical ForA fluorescence intensities were determined by two custom-built macros for ImageJ software and protocols operated in Excel. The radial displacement of the cell edge between the time point *t* and a former time point *t−1* was measured as follows: the cell boundaries of both time points were manually outlined from confocal fluorescence images as two pixel-wide lines onto blank images of the same size. Using the first custom-built macro, the radial distance between the cell centroid at *t* and the outlined cell edge was automatically determined by line scans at each angle relative to the direction of movement (defined by the cell centroids at *t* and *t−1* and set as 0°, Supplementary Fig. 2a). For this purpose, the macro employs the commands ‘plot', ‘profile' and ‘rotate'. The net displacement of the cell boundary at each angle corresponds to the difference of the boundary distances at *t−1* and *t* and was calculated in Excel. ForA fluorescence intensity profiles at each angle were generated by the second macro (Supplementary Fig. 2b). The average of the three brightest pixels at the determined cortical position was extracted from the profile data by a custom-built Excel protocol. The resulting angular profiles correlating protrusion and retraction rates with ForA fluorescence intensity before, during and after repolarization are exemplarily shown in Supplementary Fig. 2c.

*Micropipette aspiration*. For the micropipette aspiration, a chamber having one open side was filled with PB buffer and mounted on a stage of an inverted Axiovert 200 microscope (Zeiss) equipped with a LD Achroplan × 40/0.6 objective (Zeiss). The position of the micropipettes was controlled by a SM 3.25 micromanipulator (Märzhäuser Wetzlar). Aspiration pressure was applied by a height-adjustable water reservoir driven by a piezoelectric magnet slider and was calibrated with 2-μm polystyrene beads (Polysciences) to obtain 0 Pa. For the experiments, glass capillaries with an inner diameter of 3.4±0.8 μm were used. After setting the pressure difference to 500 or 1,250 Pa, PB buffer washed growth-phase cells were carefully injected into the chamber, and only non-adhering cells aspirated. The aspirations were recorded by differential interference contrast (DIC) imaging using a Sensicam CCD camera (PCO) with a frame rate of at least 2 Hz. Data were analysed by Fiji software.

### Lipid co-floatation assays

1-Palmitoyl-2-oleoyl-sn-glycero-3-phosphatidylcholine (POPC), 1-palmitoyl-2-oleoyl-sn-glycero-3-phosphatidylethanolamine (POPE), 1-palmitoyl-2-oleoyl-sn-glycero-3-phosphatidylserine (POPS), phosphatidyl-inositol-(3)-phosphate, phosphatidyl-inositol-(4)-phosphate, phosphatidyl-inositol-(3,5)-bisphosphate, PI(4,5)P_2_ and PI(3,4,5)P_3_ were purchased from Avanti Polar Lipids. Lipid compositions were POPC:POPE:POPS at a ratio of 6:2:2 for the carrier lipids and POPC:POPE:POPS:PIPx at a ratio of 5:2:2:1. Lipids in desired concentrations were mixed and then dried under a stream of nitrogen. The lipids were subsequently maintained under reduced pressure for at least 3 h, and subsequently dissolved in 20 mM HEPES buffer, pH 7.4, 100 mM NaCl and 0.3 M sucrose with occasional gentle vortexing for 1 h at room temperature to form liposomes. For visualization, liposomes were labelled with 5% rhodamine B-conjugated PE (Avanti polar lipids). A mixture of GST-ForA-C2 (1.72 μM) and liposomes (total lipid 166.67 μM) was incubated at room temperature for 15 min. Samples were then brought to 30% sucrose in HEPES buffer by gentle mixing (final volume 250 μl) and overlaid with 200 μl each containing either 25% sucrose or 0% sucrose solution in the above described HEPES buffer. The samples were centrifuged at 50,000 r.p.m. for 30 min at 4 °C with a Beckman Optima Max ultracentrifuge using a TLS55 rotor. Fractions of 100 μl were collected from top (fraction 1) to bottom (fraction 4), and 25-μl samples analysed by SDS–polyacrylamide gel electrophoresis and Coomassie blue staining. Intensities of protein bands in fraction 2 were quantified by densitometry using the Quantity One software (Bio-Rad), and are expressed as percentage of the total protein.

### GTPase activation assays

For Rac1-GTP-binding assays, 200 μg of MBP fused to PAK-CRIB was immobilized on amylose high-flow resin (NEB). Cells grown in 10-cm-diameter dishes were harvested by centrifugation, washed twice with cold PB buffer (17 mM Na-K-phosphate, pH 6.0) and lysed with 1 ml of ice-cold lysis buffer (25 mM Tris, pH 7.5, 10 mM MgCl_2_, 50 mM NaCl, 2 mM EGTA, 2 mM DTT, 1% *n*-octylpolyoxyethylene (Bachem), 2 mM benzamidine, bestatin (0.5 mg ml^−1^), and pepstatin, antipain and leupeptin (each 1 mg ml^−1^). After centrifugation at 15,000*g* for 10 min, 900 μl of the lysates each were added to the beads and incubated for 60 min at 4 °C on a rotary wheel. After the beads were washed three times with wash buffer (25 mM Tris, pH 7.5, 10 mM MgCl_2_, 50 mM NaCl, 2 mM DTT and 0.1% *n*-octylpolyoxyethylene), the precipitates were analysed by immunoblotting with polyclonal anti-Rac1 antibodies. For quantification of relative Rac1-GTP levels, band intensities were measured by densitometry and analysed using ImageJ software. Band intensities of active Rac1 from four independent experiments were averaged and normalized to wild-type levels.

### Statistical analysis

Statistical analysis was performed using SigmaPlot 11.2 software (Systat Software Inc.). Statistical significance of differences between non-normally distributed populations was determined by the Mann–Whitney *U*-test. When data fulfilled the criteria of normality (Shapiro–Wilk test) and equal variance (Levene's test), statistical differences were analysed with a two-tailed, unpaired Student's *t*-test. Statistical differences are reported as *P≤0.05, **P≤0.01, ***P≤0.001 and NS as not significant.

## Additional information

**How to cite this article:** Ramalingam, N. *et al*. A resilient formin-derived cortical actin meshwork in the rear drives actomyosin-based motility in 2D confinement. *Nat. Commun*. 6:8496 doi: 10.1038/ncomms9496 (2015).

## Supplementary Material

Supplementary Information Supplementary Figures 1-6 and Supplementary Table 1

Supplementary Movie 1 GFP-tagged full-length ForA is autoinhibited and entirely cytoplasmic. Reconstituted forA- growth-phase cells expressing GFP-ForA-FL were imaged on 3 cm glass-bottom dishes (Matek) in PB buffer using 488 nm laser excitation and pseudo-phase contrast. Time is min:s. Scale bar, 10 μm.

Supplementary Movie 2 GFP-tagged constitutively active ForA accumulates in the trailing edge of migrating cells. Reconstituted forA- growth-phase cells expressing GFP-ForA-ΔDAD were imaged as above using the 488 nm laser excitation. Fluorescence intensity distributions of 8-bit grey scale images are displayed in pseudo-colour in direction of the z-axis to illustrate the highly polarised distribution of the formin in the rear of the migrating cell. Time is min:s. Scale bar, 10 μm.

Supplementary Movie 3 Constitutively active ForA is excluded from nascent protrusions. Reconstituted forA- growth-phase cells expressing GFP-ForAΔDAD were imaged as above using 488 nm laser excitation. Fluorescence intensity distributions of 8-bit grey scale images are displayed in pseudo-colour. The spontaneous attempt to form a new front in the rear (white arrow head) - which in this case eventually failed - was associated with concomitant disappearance of the formin. This demonstrates that the formation of migratory protrusions and presence of the active formin are mutually exclusive. Time is min:s. Scale bar, 10 μm.

Supplementary Movie 4 Dynamic relocalisation of active ForA in front-to-tail transitions of turning cells. Reconstituted forA- growth-phase cells expressing GFP-ForAΔDAD were imaged as above. Successful establishment of a new front in sharply turning cells leads to disappearance of the formin from the former rear and appearance at the prospective trailing edge. Time is min:s. Scale bar: 10 μm.

Supplementary Movie 5 ForA nucleates actin polymerisation. Time-lapse recordings of single actin filaments by in vitro TIRF-microscopy. The nucleation of actin filaments (1.0 μM actin, 10% Atto488-labelled) in the absence or presence of 10 or 100 nM ForA-C, respectively was visualised in an area of 80 × 80 μm. Substantially more filaments were formed in the presence of ForA. Time is min:s. Scale bar, 10 μm

Supplementary Movie 6 ForA promotes actin filament elongation in the presence of profilin. Time-lapse recordings of single actin filaments by in vitro TIRF-microscopy. The elongation of actin filaments (1.0 μM actin, 10% Atto488-labelled) and 5 μM PFNI in the absence or presence of 10 nM ForA-C was visualised in an area of 80 × 80 μm. Elongating filament barbed ends are highlighted with white arrow bars. Time is min:s. Scale bar, 10 μm.

Supplementary Movie 7 Loss of ForA promotes random cell migration in unconfined environments. Migration of wild-type and forA- cells on a glass surface. The cells were recorded by phase-contrast imaging in PB buffer using 10 x magnification. Time is min:s. Scale bar: 10 μm.

Supplementary Movie 8 Loss of ForA inhibits cell migration in 2D-confined environments. Migration of compressed wild-type and forA- cells. The cells were recorded by phase-contrast imaging in PB buffer using 10 x magnification. Time is min:s. Scale bar, 10 μm.

Supplementary Movie 9 ForA is required for cortical integrity. Aspiration of wild-type and mutant cells at 500 Pa or 1250 Pa in PB buffer. At a constant suction pressure of 500 Pa the initial projection length of forA- cells is larger as opposed to wild-type cells, but both cell lines are able to withstand the suction pressure, while the shown iqgp1-/iqgp2- and ctxI-/ctxII- mutant cells were completely aspirated. At a suction pressure of 1250 Pa forA- cells were entirely aspirated into the micropipette whereas wild-type cells were still able to resist this suction pressure. Time is min:s. Scale bar, 20 μm.

Supplementary Movie 10 Elimination of ForA in mhcA--mutants does not affect cell migration in unconfined environments. Migration of mhcA- and mhcA-/forA- cells on a glass surface. The cells were recorded by phase-contrast imaging in PB buffer using 10 x magnification. Time is min:s. Scale bar, 10 μm.

Supplementary Movie 11 mhcA- and mhcA-/forA- cells are immotile in 2D-confinement. The behaviour of mhcA- and mhcA-/forA- cells compressed under agar was recorded by phase-contrast imaging in PB buffer using 10 x magnification. Note the inability of both cell lines to migrate in 2D-confinement. In contrast to mhcA- cells, however, the mhcA-/forA- cells formed numerous protrusions around their cell periphery. Time is min:s. Scale bar, 25 μm.

Supplementary Movie 12 Accumulation of GFP-tagged myosin II to the rear of cells migrating under agar. Wild-type derived cells expressing the heavy chain of myosin II fused to GFP were imaged on 3 cm glass-bottom dishes in PB buffer compressed under agar using 488 nm laser excitation and pseudo-phase contrast. Time is min:s. Scale bar, 10 μm.

Supplementary Movie 13 Loss of ForA impairs cell migration in 2D-confined environments due to extensive blebbing in the rear. forA- cells expressing the heavy chain of myosin II fused to GFP were imaged on 3 cm glass-bottom dishes in PB buffer compressed under agar using 488 nm laser excitation and pseudo-phase contrast. Time is min:s. Scale bar, 10 μm.

Supplementary Movie 14 Impaired migration of forA- cells in 2D-confined environments. This Video refers to Videos 12 and 13 to illustrate dynamic behaviour of cell outlines (white) in relation to the cell centroids (cyan dots). Scale bar, 10 μm.

Supplementary Movie 15 Active mDia1 accumulates at the rear cortex of migrating B16-F1 cells. GFP-mDia1ΔDAD expressing B16-F1 cells were imaged on 3 cm glass-bottom dishes coated with laminin in a heated microincubator using epifluorescence and phase-contrast microscopy and 40 x magnification. Note the highly polarised distribution of the formin in the rear cortex of the migrating cell. Time is min:s. Scale bar, 10 μm.
