# Supplementary material for: A resilient formin-derived cortical actin meshwork in the rear drives actomyosin-based motility in 2D confinement
Source: Nat Commun. 2015 Sep 29;6:8496. doi: 10.1038/ncomms9496 (PMC4598863; doi:10.1038/ncomms9496)
Supplement: Supplementary Information — Supplementary Figures 1-6 and Supplementary Table 1 [file ncomms9496-s1.pdf]

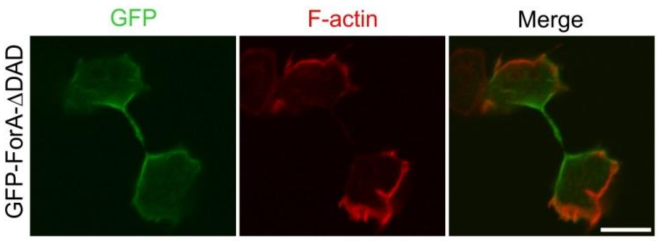


**Supplementary Figure 1: Localisation of active ForA in mitotic cells.** Reconstituted *forA*⁻ cells expressing constitutively active ForA tagged with GFP were fixed and stained with Rhodamine-phalloidin for filamentous actin (red). The GFP signal was enhanced with Alexa488-conjugated nanobodies (green). Confocal sections are shown. Active ForA accumulated in the cleavage furrow of dividing cells (bottom). Scale bar, 10 µm.

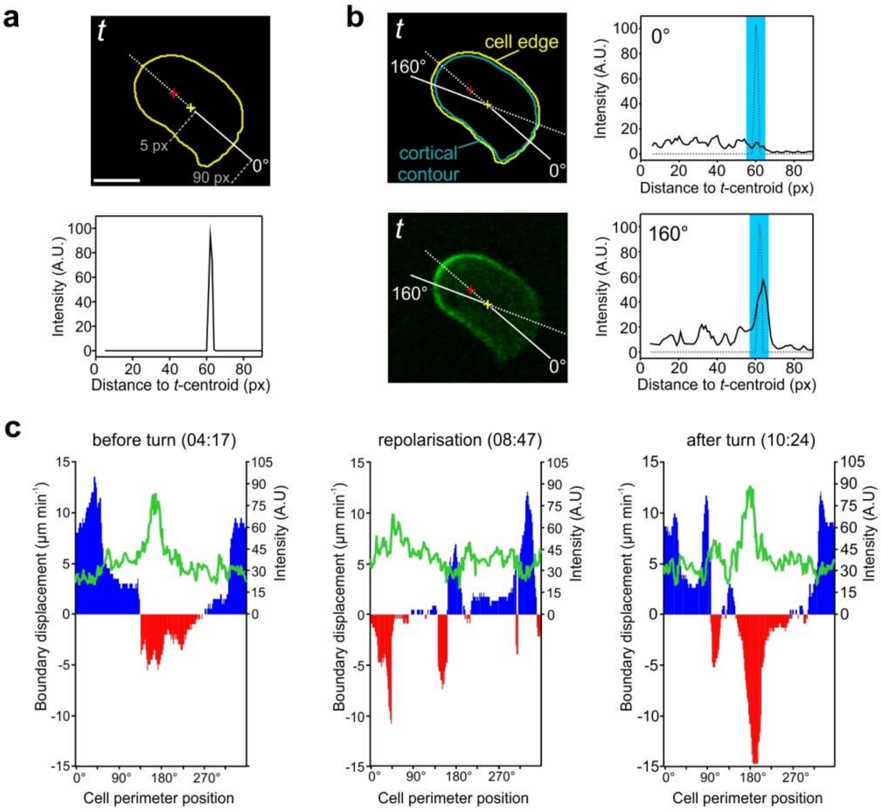


**Supplementary Figure 2: Determination of protrusion/retraction rates and cortical ForA intensity. (a)** Principle of semi-automated determination of the radial boundary distance from the *t*-centroid by the first custom-built macro. The outlined cell edge at time point *t* is displayed in yellow. Crosses indicate centroids at time points *t* (yellow) and *t-1* (red) and define the 0° degree axis. During the line scan, pixel intensities were measured along an 85 pixel (px) long solid section of a line that was manually centred on the *t*-centroid. The diagram shows the resulting pixel intensity profile. The macro automatically performs the line scan, identifies the maximum of the profile and records the distance from the *t*-

centroid (62 px in this case). Subsequently, the line is automatically rotated clockwise in one-degree steps in between the following line scans. Scale bar, 10 µm. **(b)** Principle of the automated quantification of the cortical GFP-ForAΔDAD intensity. Examples are shown for 0° and 160°. The radial distance between the outlined cortical contour and the *t*-centroid is determined by the first macro. The second macro records the fluorescence intensity along the 85 px line section. Diagrams show fluorescence line scans (black, solid line) and cortical contour line scans (black, dashed line) at *t* for the indicated angle. Grey boxes depict a ± 5 px wide region at the determined cortex position. Within this region, the three brightest fluorescence pixels were averaged. (**c**) Angular profiles of protrusion/retraction rates and cortical ForA intensity (green) prior, during and after repolarisation of the cell shown in Supplementary Video 4 and Fig. 1c. Protrusion (blue) and retraction (red) rates were determined between the frames as indicated in Fig. 1c and at time points 03.47, 08:15 and 09:51, respectively. Note, that ForA accumulated at regions with high retraction rates during the migration (before and after repolarisation), while the formin was more equally distributed during the repolarisation phase of the cell.

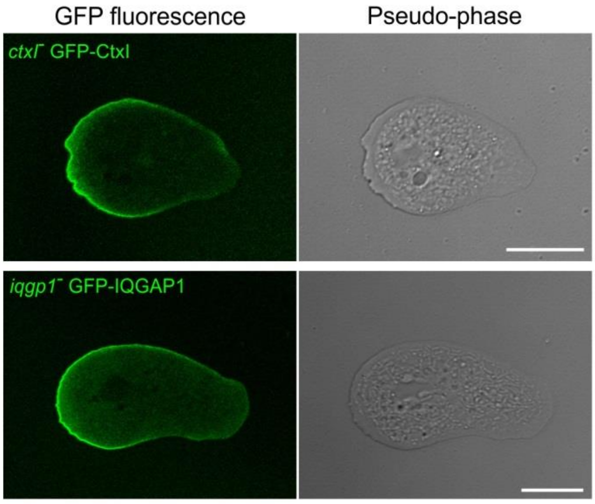


**Supplementary Figure 3:** Increased accumulation of GFP-tagged CtxI and IQGAP1 in the rear after compression under agar. Reconstituted *ctxI⁻* and *iqgp1⁻* cells expressing either GFP-tagged CtxI or IQGAP1 were imaged on 3 cm glass-bottom dishes in PB buffer using 488 nm laser excitation and pseudo-phase contrast. Note prominent accumulation of the fusion proteins in the posterior cortex and diminished cytosolic localisation as compared to freely moving cells (see Fig. 2a). Scale bars, 10 µm.

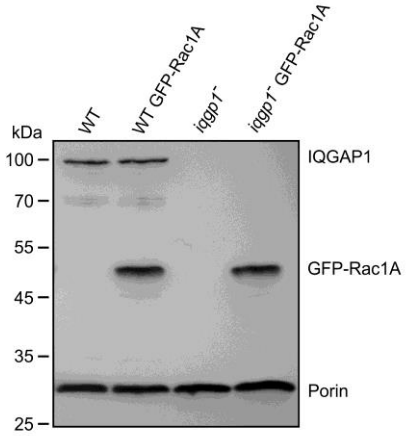


**Supplementary Figure 4: Overexpression of Rac1 in wild-type and *iqgp1*⁻ cells.** Equal amounts of total cellular proteins of the wild-type and *iqgp1*⁻ cells expressing the GFP-tagged wild type form of Rac1A were subjected to SDS-PAGE, blotted onto nitrocellulose, and labelled with anti-IQGAP1 anti-GFP. Porin staining served as an internal loading control. The immunoblot shows that both cell lines express similar amount of GFP-tagged Rac1A.

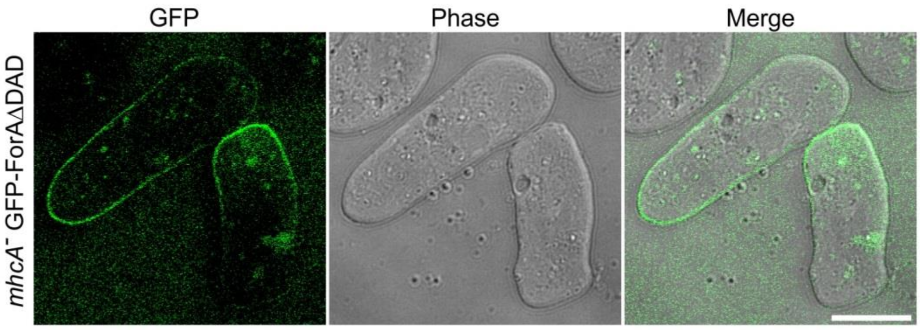


**Supplementary Figure 5: Localisation of ForA does not require myosin II.** Reconstituted *mhcA*⁻ cells expressing GFP-ForAΔDAD were imaged on 3 cm glass-bottom dishes in PB buffer under agar using 488 nm laser excitation and pseudo-phase contrast. Note prominent accumulation of the active formin in the rear cortex. Scale bar, 10 µm.

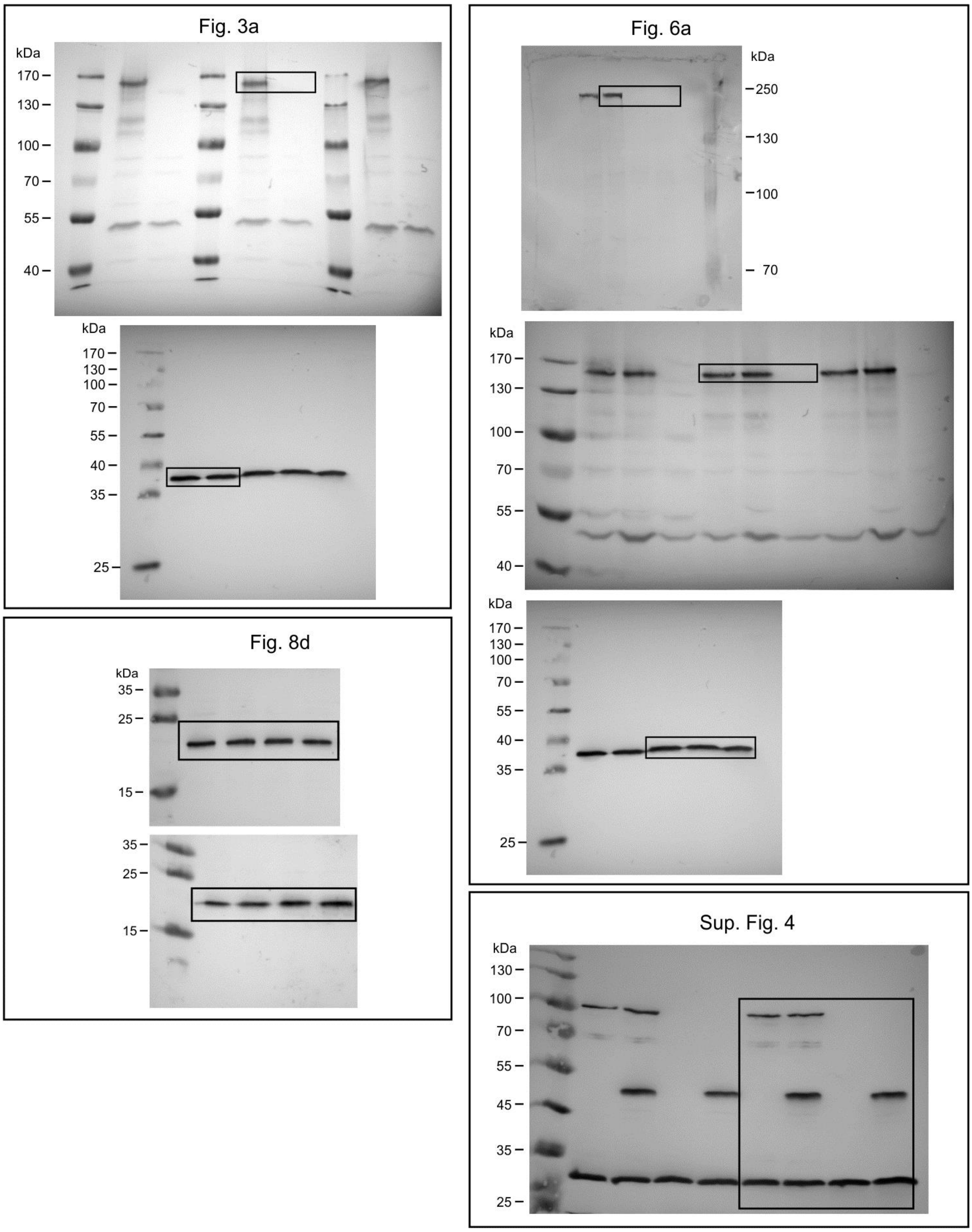


**Supplementary Figure 6: Uncropped images of blots.**

| Primers | Sequence | Orientation |
|---|---|---|
| ***Dictyostelium constructs*** | | |
| **ForA-KO 4x** | | |
| ForA-5'-KO-BU | 5'-CGCGGGATCCGCATGGCCGATAAATTGTACCAAATTAAATTG-3' | forward |
| ForA-5'-KO-PstD | 5'-CGCCTGCAGAACCACGGGAACGTAAGGCTACAC-3' | reverse |
| ForA-3'-KO-H3U | 5'-CGCAAGCTTGGTACTGCTAAACCAGATTCCG-3' | forward |
| ForA-3'-KO-SD | 5'-CGCGTCGACGGAATTCATTGATATTATTGATGT-3' | reverse |
| | | |
| **ForA-N (1-647)** | | |
| ForA-BU | 5'-GCGGGGATCCTCATGGCCGATAAATTGTACCAAATTAAA-3' | forward |
| ForA-647-SD | 5'-GCGGTCGACTTAACTAGTTGAAGCAGCAGCGGAATC-3' | reverse |
| | | |
| **ForA-C (647-1218)** | | |
| ForA-647-SpeU | 5'-TCCGCTGCTGCTTCAACTAGTGTTGCTCCA-3' | forward |
| ForA-1218-SD | 5'-GCGGTCGACTTACAAATCAATTGGTTCTATAGTCGA-3' | reverse |
| | | |
| **ForAΔDAD (1-1138)** | | |
| ForA-BU | 5'-GCGGGGATCCTCATGGCCGATAAATTGTACCAAATTAAA-3' | forward |
| ForA-1138-SD | 5'-GCGGTCGACTTATTGTTCATTCTCTTTGGTAGCTTTATC-3' | reverse |
| | | |
| **ForAΔC2ΔDAD (91-1138)** | | |
| ForA-91-BU | 5'-GCGGGGATCCTCACTTATCAATTACCAGAATCATTAATT-3' | forward |
| ForA-1138-SD | 5'-GCGGTCGACTTATTGTTCATTCTCTTTGGTAGCTTTATC-3' | reverse |
| | | |
| **ForAC2 (1-99)** | | |
| ForA-BU | 5'-GCGGGGATCCTCATGGCCGATAAATTGTACCAAATTAAA-3' | forward |
| ForA-C2-99-SD | 5'-CGCGTCGACTTAATTAATGATTCTGGTAATTG-3' | reverse |
| | | |
| ***E. coli constructs*** | | |
| **ForA-FH3 (139-539)** | | |
| ForA-139-BU | 5'-CCAGGATCCGAAGAAAAGAAAAGAC-3' | forward |
| ForA-539-SD | 5'-CGCGTCGACTTAACCAATATTCTTGTCACGTA-3' | reverse |
| | | |
| **ForA-FH3L (aa 91-647)** | | |
| ForA-91-BU | 5'-GCGGGATCCACTTATCAATTACCAGAATCATTAATT-3' | forward |
| ForA-647-SD | 5'-GCGGTCGACTTAACTAGTTGAAGCAGCAGCGGAATC-3' | reverse |
| | | |
| **ForA-C (635-1218)** | | |
| ForA-C-635-BU | 5'-GCGGGATCCGGTACTGCTAAACCAGATTCC-3' | forward |
| ForA-C-1218-SD | 5'-CGCGTCGACTTACAAATCAATTGGTTCTATAGTCGA-3' | reverse |
| | | |
| **ForA-C2 (1-99)** | | |
| ForA-C2-6P-BU | 5'-GCGGGATCCATGGCCGATAAATTGTACCAAAT-3' | forward |
| ForA-C2-99-SD | 5'-CGCGTCGACTTAATTAATGATTCTGGTAATTG-3' | reverse |
| | | |
| **PAK-GBD** | | |
| PAK-CRIB-BU | 5'-CGCGGATCCTCCATCTTAGCTGGAGATAAAAC-3' | forward |
| PAK-CRIB-SD | 5'-CGCGTCGACTCAATATACAGATTTTGTGTGTTCTG-3' | reverse |

## Supplementary Table 1: Primers
